# Microliter Scale Synthesis of Luciferase‐Encapsulated Polymersomes as Artificial Organelles for Optogenetic Modulation of Cardiomyocyte Beating

**DOI:** 10.1002/advs.202200239

**Published:** 2022-07-28

**Authors:** Hyemin Kim, Jonathan Yeow, Adrian Najer, Worrapong Kit‐Anan, Richard Wang, Omar Rifaie‐Graham, Chalaisorn Thanapongpibul, Molly M. Stevens

**Affiliations:** ^1^ Department of Materials Department of Bioengineering and Institute of Biomedical Engineering Imperial College London London SW7 2AZ UK

**Keywords:** artificial organelles, bioluminescences, cardiomyocytes, nanoreactors, optogenetics, polymerization‐induced self‐assembly, polymersomes

## Abstract

Constructing artificial systems that effectively replace or supplement natural biological machinery within cells is one of the fundamental challenges underpinning bioengineering. At the sub‐cellular scale, artificial organelles (AOs) have significant potential as long‐acting biomedical implants, mimicking native organelles by conducting intracellularly compartmentalized enzymatic actions. The potency of these AOs can be heightened when judiciously combined with genetic engineering, producing highly tailorable biohybrid cellular systems. Here, the authors present a cost‐effective, microliter scale (10 µL) polymersome (PSome) synthesis based on polymerization‐induced self‐assembly for the in situ encapsulation of *Gaussia* luciferase (GLuc), as a model luminescent enzyme. These GLuc‐loaded PSomes present ideal features of AOs including enhanced enzymatic resistance to thermal, proteolytic, and intracellular stresses. To demonstrate their biomodulation potential, the intracellular luminescence of GLuc‐loaded PSomes is coupled to optogenetically engineered cardiomyocytes, allowing modulation of cardiac beating frequency through treatment with coelenterazine (CTZ) as the substrate for GLuc. The long‐term intracellular stability of the luminescent AOs allows this cardiostimulatory phenomenon to be reinitiated with fresh CTZ even after 7 days in culture. This synergistic combination of organelle‐mimicking synthetic materials with genetic engineering is therefore envisioned as a highly universal strategy for the generation of new biohybrid cellular systems displaying unique triggerable properties.

## Graphical abstract

Herein, the authors present a microliter scale synthesis of enzyme‐loaded polymersomes based on polymerization‐induced self‐assembly as a versatile approach for fabricating highly robust artificial organelles (AOs). The luminescence of encapsulated luciferase is highly resistant to thermal, proteolytic, and intracellular stresses allowing these AOs to show cardiostimulatory effects in optogenetically modified induced pluripotent stem cell‐derived cardiomyocytes even after a week in vitro.

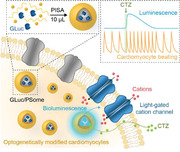

## 1 Introduction

Compartmentalization of biochemical reactions is an essential property of complex organisms and critical for the maintenance of homeostasis. In living cells, this primarily occurs within organelles that perform compartmentalized biochemical transformations and control the flow of reactants in and out of the organelle environment.^[^ ^1^ ^]^ The interplay between organelles and the intracellular environment is highly regulated and therefore a critical component in dictating regular cellular function. In the fields of nanomedicine and synthetic biology, synthetic organelle mimics known as artificial organelles (AOs) have shown particular promise as biomedical implants owing to their potential to replace or supplement intrinsic cellular functions.^[^ ^2^ ^]^ More recently, this concept has also been expanded to implement AO systems that can even add non‐native, orthogonal pathways for modulating cellular behavior.^[^ ^3^ ^]^

Ideal AOs should exhibit a number of key characteristics including: i) the ability to perform compartmentalized (bio)catalytic reactions, ii) long‐term maintenance of catalytic activity in an intracellular environment, and iii) high biocompatibility.^[^ ^4^ ^]^ In order to mimic organelle structures, AOs most commonly consist of an enzyme encapsulated within a semipermeable membrane that isolates the catalytic machinery from potential intracellular stresses such as proteases. Polymer‐based vesicles (polymersomes) as AOs are highly attractive as they can be engineered to possess greater stability (and broader chemical functionality) compared to liposomes,^[^ ^5^ ^]^ enabling them to be optimized for the provision of long‐term intracellular therapeutic activity. However, to date, polymersome (and liposomal) formulations have been limited to the incorporation of relatively inexpensive enzymes, which is largely owing to the reaction scale (milliliter scale) at which the self‐assembly must be performed.^[^ ^6^ ^]^ Recently, advances in the synthesis of polymersomes using (photoinitiated) polymerization‐induced self‐assembly (PISA) have been leveraged as an in situ chemical method to fabricate enzyme‐encapsulated polymersomes.^[^ ^7^ ^]^ In some cases, these methods have reported relatively high encapsulation efficiencies for protein cargo which could be due to the concentration at which PISA can be performed, although the role of polymer/protein interactions has yet to be comprehensively studied in this context. Advantageously, such polymersomes are reported to be intrinsically permeable to small molecules, and therefore do not require chemical modification or incorporation of membrane‐integrated porins for effective catalytic function.^[^ ^7^, ^8^ ^]^ In contrast to conventional polymersome and liposome self‐assembly techniques (such as thin‐film rehydration) which are typically performed on the milliliter scale, PISA, as a chemically driven self‐assembly process, has potential to be downscaled (e.g., to microliter volumes) to allow for otherwise cost‐prohibitive enzymes to be employed, and thereby significantly broaden the scope and feasibility of the AO approach in bioengineering and therapeutics.

Distinct from synthetic AO implants, genetic manipulation represents a bioengineering approach for artificial modulation of cellular function. This is seen in the rapidly developing field of optogenetics where transgenically modified mammalian cells with light‐activated ion channels are used to allow for light‐based modulation of cellular function.^[^ ^9^ ^]^ For example, excitable cell types such as neural or cardiac cells can be genetically modified to express channelrhodopsin‐2 (ChR2), a membrane bound retinylidene protein that triggers cation influx under blue light, allowing cells to undergo light‐induced depolarization.^[^ ^10^ ^]^ In cardiomyocytes, this light‐triggered depolarization can then also initiate the key cardiac function of mechanical contraction due to calcium‐driven myosin–actin interactions.^[^ ^11^ ^]^ Optogenetically engineered cardiomyocytes, therefore, provide unique opportunities in novel therapies for cardiac diseases as well as broader applications, e.g., in cell‐driven biohybrid soft robotic devices.^[^ ^12^ ^]^

**Scheme 1:**
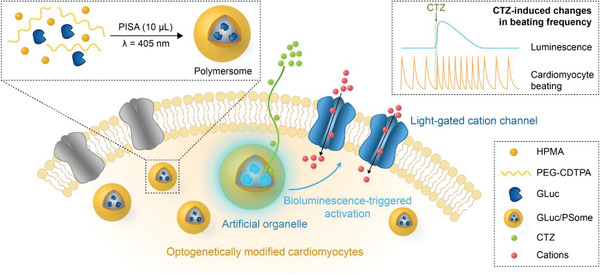
Overview of the proposed AO system for modulating cardiomyocyte beating. PISA‐derived GLuc/PSomes as AOs are taken up into iPSC‐CMs and generate blue luminescence when the cells are treated with CTZ. This blue light can activate ChR2‐transduced iPSC‐CMs, triggering cation influx, and an increase in beating frequency.

Herein, we combine synthetic polymer self‐assembly techniques with genetic engineering by developing a downscaled microliter volume PISA synthesis of polymersome‐based AOs that can stimulate the beating of cardiomyocytes derived from optogenetically engineered induced pluripotent stem cells (iPSCs) (**Scheme** **1**). To couple AO activity with the beating of iPSC‐derived cardiomyocytes (iPSC‐CMs), *Gaussia* luciferase (GLuc) was encapsulated within the AO as an intracellular bioluminescent light source capable of activating genetically modified ChR2‐transduced iPSC‐CMs. Advantageously, the bioluminescence of GLuc within this AO system also readily enables direct monitoring of AO activity within live cells and over prolonged time periods (e.g., days) without the need for specific assays to detect enzymatic turnover. Since GLuc is a relatively expensive enzyme (>1000 USD per mg), we developed a novel protocol enabling polymersome synthesis to be scaled down to a 10 µL volume (≈10 µg GLuc per synthesis) greatly improving the cost efficiency and viability of AO syntheses. Importantly, these PISA‐derived GLuc‐encapsulated polymersomes (GLuc/PSomes) are natively permeable to the enzyme substrate, coelenterazine (CTZ), without the need for insertion of membrane permeabilizing moieties. Furthermore, when taken up into iPSC‐CMs, GLuc/PSomes displayed significantly prolonged retention of intracellular bioluminescence activity compared to the free enzyme. In the presence of CTZ, GLuc/PSomes internalized within the optogenetic iPSC‐CMs produced blue luminescence to stimulate cell‐membrane bound ChR2 and increase the frequency of spontaneous beating. This proof‐of‐concept demonstrates a unique and completely nonnative AO‐mediated cascade for cardiomyocyte stimulation that is also highly specific, since both CTZ and light are generally bioorthogonal species. Importantly, this CTZ‐induced cardiostimulatory activity could be re‐activated with fresh CTZ even after 7 days in vitro (well beyond the typical in vitro timeframes for AO studies), highlighting the significant potential of these GLuc/PSomes as potent, long‐acting AOs. We envision that this unique combination of low‐volume AO synthesis with genetic engineering will open new avenues toward triggerable biohybrid cellular systems, by incorporating previously cost‐prohibitive enzymes for modulation of a broad range of natural and non‐natural cellular behaviors.

## 2 Results and Discussion

**Figure 1:**
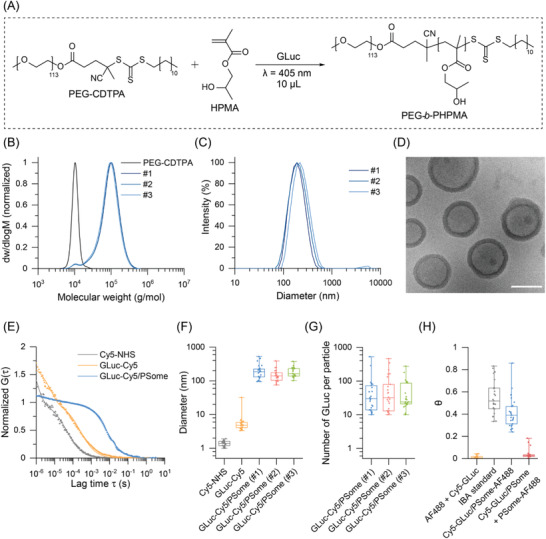
Synthesis and characterization of GLuc‐loaded polymersomes using microliter volume PISA. A) Chemical scheme showing the downscaled synthesis of PEG‐*b*‐PHPMA amphiphilic block copolymer under aqueous photoiniferter‐based PISA conditions at a reaction volume of 10 µL. B) Molecular weight distributions of PEG‐CDTPA and unpurified PEG‐*b*‐PHPMA block copolymers synthesized in the presence of GLuc (*n* = 3, synthetic replicates). C) Average DLS intensity‐based distribution of purified GLuc/PSomes (data represent the mean of three repeated measurements for three synthetic replicates). D) Representative cryo‐EM image of purified GLuc/PSomes. Scale bar: 200 nm. E) Normalized FCS autocorrelation curves (solid lines) for Cy5‐NHS, GLuc‐Cy5, and GLuc‐Cy5/PSome (average curves of *n* = 25 individual measurements). Symbols represent raw data and lines represent fitted curves. F) Hydrodynamic diameter distributions calculated from FCS autocorrelation curves for Cy5‐NHS, GLuc‐Cy5, and synthetic triplicates of GLuc‐Cy5/PSome. G) Distribution of encapsulated GLuc molecules per PSome based on a GLuc‐Cy5:GLuc loading ratio of 1:9 (synthetic triplicates). H) FCCS‐derived relative cross‐correlation amplitudes (*θ*) for dual labeled GLuc‐Cy5/PSome‐AF488 (blue), IBA standard (gray) as well as physical mixtures of AF488 with GLuc‐Cy5 (yellow) and GLuc‐Cy5/PSome with PSome‐AF488 (red) (*n* = 25 individual measurements). Box plots: center line, median; box limits, upper and lower quartiles; whiskers, minimum and maximum values.

One common limitation of most polymersome syntheses is the relatively large scale at which self‐assembly techniques such as thin‐film rehydration and solvent exchange must be performed (typically milliliter scale), which necessitates the use of large quantities of therapeutic enzymes for encapsulation (or lowered enzyme concentrations). In the case of PISA, which is primarily based on a form of radical polymerization known as reversible addition‐fragmentation chain transfer (RAFT) polymerization,^[^ ^13^ ^]^ oxygen sensitivity likewise limits the downscaling of such syntheses below the milliliter scale since physical removal of oxygen usually requires gas‐tight (sealed) reaction vessels.^[^ ^14^ ^]^ As an alternative to using oxidase enzymes to perform deoxygenation, which would undesirably contaminate the polymersomes by co‐encapsulation,^[^ ^15^ ^]^ we overcame the issue of oxygen inhibition in PISA via the simpler approach of “polymerizing‐through” oxygen^[^ ^14^, ^16^ ^]^ and physically limiting the diffusion of atmospheric oxygen with mineral oil.^[^ ^17^ ^]^ Polymersome synthesis using PISA was based upon the chain extension of 4‐cyano‐4‐[(dodecylsulfanylthiocarbonyl)sulfanyl]pentanoic acid‐modified poly(ethylene glycol) (PEG‐CDTPA) with 2‐hydroxypropyl methacrylate (HPMA) under aqueous conditions (**Figure** **1**A). The polymerization was initiated using the photoiniferter method, whereby visible light can be used to directly generate radicals by photolysis of the RAFT agent, simplifying the reaction complexity by removing the need for catalyst or initiator.^[^ ^18^ ^]^ Advantageously, this approach has a high rate of radical generation and, by employing visible light, has greater protein compatibility compared to thermal initiation techniques. In order to strike a balance between minimizing GLuc wastage while still generating sufficient quantities of material for characterization, we targeted a reaction volume of 10 µL in the presence of GLuc at a concentration of 1 mg mL^–1^. Since physical deoxygenation techniques (e.g., nitrogen sparging) which have been employed in previous PISA approaches are extremely difficult to implement at these microliter reaction volumes, an optimized protocol was developed whereby reactions were conducted in a 1536‐well microtiter plate (in triplicate) which has capillary‐like wells that minimize the surface area exposed to atmospheric oxygen. To physically inhibit oxygen ingress and prevent evaporation, 2.5 µL of mineral oil was added on top of each well. After 3 h of irradiation with 405 nm light (*I* = 10 mW cm^–2^), ^1^H NMR indicated that relatively high HPMA conversions (77 ± 3%, *n* = 3) were achieved despite the lack of prior deoxygenation. Shifts in the gel permeation chromatography‐derived molecular weight chromatograms of the crude reaction mixture indicated successful chain extension to form the desired poly(ethylene glycol)‐*b*‐poly(2‐hydroxypropyl methacrylate) (PEG‐*b*‐PHPMA) amphiphilic block copolymer as well as excellent synthetic reproducibility across three replicates (Figure 1B). The presence of a minor low molecular weight peak at approximately the same retention time as PEG‐CDTPA suggested the presence of dead polymer chains terminated by oxygen in the early stages of the polymerization. Purification of the crude reaction mixture was conveniently performed by repeated cycles of centrifugation and resuspension, enabling efficient removal of residual monomer, unencapsulated GLuc, mineral oil as well as residual hydrophilic chains due to their solubility in PBS (Figure S1, Supporting Information). Importantly, this purification process based on centrifugation is highly attractive for microliter volume syntheses as it provides a significantly simplified and fast purification process compared to preparative size exclusion chromatography, which leads to undesirable dilution of nanoparticle solutions and cannot be as easily translated to a high throughput format. Dynamic light scattering (DLS) indicated the formation of nanoparticles with a hydrodynamic diameter of 231 ± 41 nm and polydispersity index of 0.13 ± 0.02 (Figure 1C) with cryogenic transmission electron microscopy (cryo‐EM) confirming the formation of hollow vesicle (polymersome) structures (Figure 1D and Figure S2A, Supporting Information). Similar results were obtained in the absence of GLuc suggesting minimal role of the enzyme in the self‐assembly process (Figures S2B and S3, Supporting Information). Finally, in order to demonstrate the versatility and simplicity of this deoxygenation‐free PISA approach for the synthesis of cargo‐loaded PSomes, we also successfully scaled up the reaction volume to 100 µL by translating the synthesis to a standard 384‐well plate (Figure S4, Supporting Information). Importantly, this provides flexibility in terms of the desired PSome application, since larger quantities of material may be desirable for different enzymatic systems. Although a significant focus of this study is to demonstrate the economic advantages of a downscaled microliter volume AO synthesis within the academic arena, it should be noted that the PISA process is also highly conducive for production at more industrially relevant synthetic scales. Compared to conventional PSome‐forming approaches such as thin‐film rehydration which can only be performed under relatively dilute conditions (typically <5 mg mL^–1^), aqueous PISA syntheses (with or without deoxygenation) can produce nanoparticles up to 250 mg mL^–1^ and have been employed to produce polymersomes at the multigram scale (>30 g) in both batch^[^ ^19^ ^]^ and continuous flow formats.^[^ ^20^ ^]^

To further characterize these GLuc/PSomes, we applied fluorescence correlation spectroscopy (FCS), a highly sensitive single particle spectroscopic technique that enables diffusion‐based measurements of fluorescently labeled species in solution.^[^ ^21^ ^]^ By including a minor population of Cy5‐labeled GLuc (GLuc‐Cy5) within the PSome, FCS revealed that the diffusion of GLuc‐Cy5 after the PISA process (GLuc‐Cy5/PSomes) was significantly slower compared to the free enzyme, indicating an association of the protein with the larger, slower diffusing nanoparticles (Figure 1E). Importantly, strong reproducibility was observed across three replicate syntheses with an average of 29 ± 4 GLuc proteins per PSome (Figure 1F,G). To confirm the encapsulation of GLuc within the PSomes, we applied a related form of FCS known as fluorescence cross‐correlation spectroscopy (FCCS) which measures the degree to which the diffusion of two separate fluorescently labeled species are correlated when moving through a confocal volume.^[^ ^22^ ^]^ To avoid photomasking interactions of the polymerization light source (405 nm) with blue light absorbing dyes, a post‐modification labeling approach for the polymersome was developed whereby GLuc‐Cy5/PSome was first synthesized in the presence of a small amount of the azide functional monomer, 3‐azido‐2‐hydroxypropyl methacrylate (AzHPMA). After purification, the azide side chains on the polymersome were subsequently labeled with AF488‐DBCO using a bioorthogonal Cu‐free click reaction to afford the dual‐labeled GLuc‐Cy5/PSome‐AF488. FCCS revealed a high correlation (*θ*) between the diffusion of the AF488‐labeled polymersome and Cy5‐labeled GLuc relative to the FCCS standard (IBA Life Sciences) (Figure 1H and Figure S5, Supporting Information). Importantly, physical mixtures of dyes and separately labeled polymersomes (GLuc‐Cy5/PSome and PSome‐AF488) did not result in co‐diffusion suggesting an encapsulation mechanism for GLuc within the dual‐labeled GLuc‐Cy5/PSome‐AF488.

To quantify the encapsulation efficiency of GLuc within the PSome, we employed a modified Micro BCA protein assay using sodium dodecyl sulfate to first lyse the polymersome, removing light scattering effects from the colorimetric assay and releasing the encapsulated GLuc. GLuc/PSomes were calculated to have an encapsulation efficiency of 7.4 ± 1.2% with empty polymersomes producing negligible bicinchoninic acid (BCA) signal (Figure S6, Supporting Information). This assay was also used to verify the purification of the GLuc/PSomes with no GLuc detected in the supernatant after three centrifugation/resuspension cycles. Such encapsulation efficiencies are in line with the PISA literature,^[^ ^5c^ ^]^ although improvements could potentially be achieved by increasing the initial protein feed concentration^[^ ^7d^ ^]^ or by integrating judicious polymer^[^ ^23^ ^]^ or protein^[^ ^6a^ ^]^ engineering.

**Figure 2:**
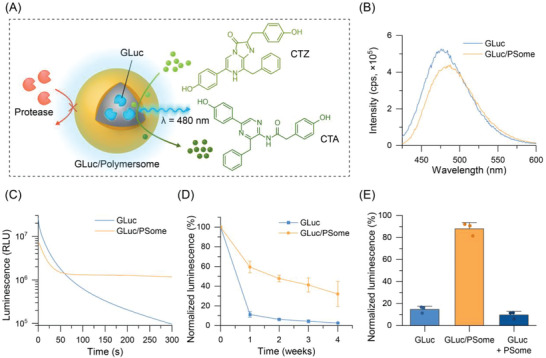
Luminescence and stability of GLuc/PSomes. A) Schematic illustration demonstrating enzymatic conversion of CTZ to coelenteramide (CTA) and concomitant luminescence emission from GLuc/PSomes. The polymersome is proposed to act as a size selective membrane leading to enhanced resistance to enzymatic degradation. B) Representative luminescence output spectra of GLuc and GLuc/PSome when treated with CTZ. C) Luminescence kinetics of GLuc and GLuc/PSomes when treated with CTZ. D) Retention of luminescence of GLuc or GLuc/PSomes after prolonged incubation at 37 °C in DPBS. E) Luminescence of GLuc, GLuc/PSomes, or empty PSomes spiked with GLuc after incubation with elastase (0.5 µg mL^–1^) for 24 h. For stability studies, the maximum luminescence intensity from the first 5 s after adding CTZ was used for quantification, and retention of luminescence was normalized relative to the luminescence output at *t* = 0 (mean ± SD, *n* = 3, technical replicates). All luminescence experiments were run at an effective GLuc concentration of 100 ng mL^–1^.

The activity of GLuc/PSomes can be conveniently monitored via the luminescent output generated by enzymatic oxidation of CTZ (**Figure** **2**A). In the presence of CTZ, GLuc/PSomes produced luminescence characteristic of CTZ oxidation with a broad emission band centered at *λ* = 480 nm, confirming the semipermeable nature of our PSomes (Figure 2B). However, in contrast to free GLuc, which possessed characteristic “flash” type kinetics, the luminescence of the GLuc encapsulated within the polymersome was significantly longer lived (on the order of hours) (Figure 2C and Figure S7, Supporting information). This effect was attributed to the effects of the polymersome membrane on the diffusion of substrates into the enzyme active site as previously reported by others.^[^ ^8^, ^24^ ^]^ This permeability effect on the observed GLuc kinetics has also been observed in nonnatively permeable polymersomes incorporating outer membrane protein F (OmpF) as a size‐selective channel protein.^[^ ^25^ ^]^ Apart from modulation of the enzyme kinetics, the encapsulation of GLuc within the PSome also provided an increased resistance to denaturing environmental factors such as thermal stress. When GLuc/PSomes were incubated at 37 °C in Dulbecco's phosphate‐buffered saline (DPBS), they retained up to 32.1 ± 12.8% of their original luminescence after 1 month, whereas the free GLuc retained only 2.6 ± 0.2% of its activity under the same conditions (Figure 2D). Further, the polymersome membrane was also capable of protecting the encapsulated enzyme from denaturing proteases. When porcine elastase, as a model protease, was added to GLuc/PSomes and incubated for 24 h, 88.0 ± 5.7% of the original luminescence was retained, which was attributed to the size selective nature of the PHPMA membrane (Figure 2E). Under the same conditions, free GLuc or GLuc spiked into a solution of empty PSomes, lost nearly all their activity (14.6 ± 2.9% and 9.7 ± 3.1% activity retained, respectively) due to proteolytic digestion of the enzyme. This suggested that the GLuc in GLuc/PSomes is not simply bound to the PSome surface but is encapsulated, leading to greater retention of the enzymatic activity in the presence of proteases (Figure 2A). To further probe the enzymatic stability, we subjected GLuc/PSomes to repeated stimulations with CTZ (Figure S8, Supporting Information). Under these conditions, the encapsulated GLuc retained its ability to produce bioluminescence after repeated treatments with CTZ, with prolonged kinetics observed in each case due to the presence of the PSome membrane. Some loss of GLuc activity was observed after each stimulation which has previously been observed when free GLuc is subjected to repeated CTZ stimulation and has been attributed to an irreversible covalent binding event that occurs during the enzymatic transformation.^[^ ^26^ ^]^

To demonstrate the potential of GLuc/PSomes as AOs, we first confirmed their biocompatibility with iPSC‐CMs (>85% purity based on flow cytometry of cardiac troponin T (cTnT) staining, Figure S9, Supporting Information) by incubating cells with empty PSomes or GLuc/PSomes for 3 days and analyzing cell viability with the PrestoBlue metabolic assay (Figure S10, Supporting Information). Under these conditions, both empty and GLuc‐loaded PSomes were not cytotoxic up to polymer concentrations of 1600 µg mL^–1^ (corresponding to a maximum GLuc concentration of 800 ng mL^–1^) which is in line with literature reports of negligible cytotoxicity of PEG‐*b*‐PHPMA nanoparticles.^[^ ^5^, ^27^ ^]^ All subsequent cell experiments with both free and encapsulated GLuc were conducted using an effective GLuc concentration of 200 ng mL^–1^ (corresponding to a polymer concentration of 400 µg mL^–1^) as a balance between required luminescence intensity and cost effectiveness.

Since efficient cellular integration is an essential prerequisite of AO systems, we monitored the uptake and distribution of fluorescently labeled GLuc/PSomes using a combination of confocal microscopy and flow cytometry. The uptake kinetics of GLuc‐Cy5/PSomes was initially studied by flow cytometry, which revealed a steady increase of uptake over the first few hours that began to saturate toward the 24 h incubation time point (Figure S11A, Supporting Information). These uptake kinetics were also visualized on live cells using live wide field imaging which revealed the internalization of GLuc‐Cy5/PSomes into the iPSC‐CMs (Movie S1 and Figure S12, Supporting Information).

**Figure 3:**
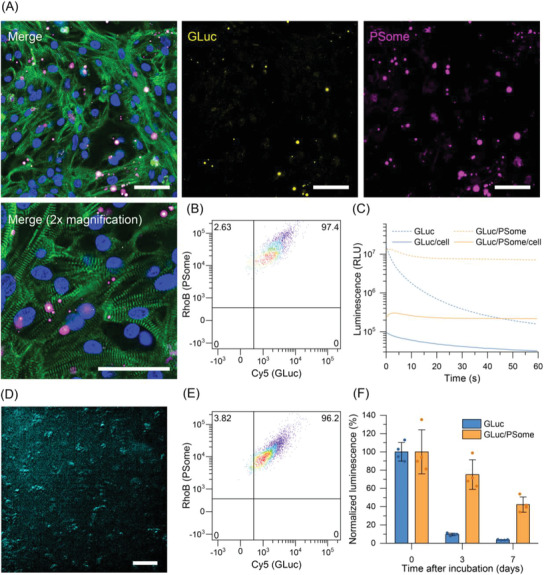
Cellular uptake and intracellular luminescence behavior of GLuc/PSomes as AOs. A) Representative confocal microscopy images and B) flow cytometry analysis of iPSC‐CMs incubated with dual‐labeled GLuc‐Cy5/PSome‐RhoB for 24 h. For confocal microscopy, the nuclei were stained with 4′,6‐diamidino‐2‐phenylindole (DAPI, blue), *α*‐actinin was stained with AF488 via a secondary antibody stain (green), GLuc with Cy5 (yellow), and the PSome with RhoB (magenta). Scale bar: 50 µm. The merged images represent the overlay of the four separate channels (the bottom image was acquired from a different region of cells at 2× higher magnification than the top images). C) Luminescence kinetics of free GLuc and GLuc/PSomes before and after their uptake into iPSC‐CMs. D) Luminescence microscopy image of iPSC‐CMs with internalized GLuc/PSomes acquired after treatment of cells with CTZ. Scale bar: 100 µm. E) Flow cytometry analysis of iPSC‐CMs cultured for 7 days after initial incubation with dual‐labeled GLuc‐Cy5/PSome‐RhoB for 24 h. F) Long‐term luminescence of free GLuc or GLuc/PSome after uptake into iPSC‐CMs. The maximum luminescence intensity from the first 10 s after adding CTZ was used for quantification, and retention of luminescence was normalized relative to the mean luminescence at day 0 (mean ± SD, *n* = 4, technical replicates).

In order to more precisely visualize the distribution of uptaken GLuc‐loaded PSomes, double‐labeled GLuc‐Cy5/PSomes were generated by performing PSome synthesis in the presence of a small amount of methacryloxyethyl thiocarbamoyl rhodamine B (RhoBMA), which undergoes radical copolymerization with HPMA into the polymer backbone to yield dual polymer and protein‐labeled GLuc‐Cy5/PSomes‐RhoB. These dual‐labeled GLuc‐Cy5/PSomes‐RhoB were incubated with iPSC‐CMs for 24 h, followed by washing with fresh medium to remove non‐uptaken PSomes and then confocal microscopy was performed on fixed, *α*‐actinin‐stained iPSC‐CMs (**Figure** **3**A). The characteristic structure of the myofibrils composed of their repeating contractile units, sarcomeres, was clearly delineated in the *α*‐actinin‐stained iPSC‐CMs, and both GLuc‐Cy5 and PSome‐RhoB signals were observed around the myofibrils. Importantly, the fluorescent signals of GLuc‐Cy5 and PSomes‐RhoB were colocalized, indicating that GLuc remained associated with the PSome even after uptake into the cells. Furthermore, wheat germ agglutinin staining, which labels glycoconjugates on cell membranes, indicated that the PSomes accumulated intracellularly rather than residing at the plasma membrane (Figure S13A, Supporting Information). This was further confirmed by the addition of Z‐stack imaging and a 3D reconstruction showing the intracellular distribution of uptaken PSomes (Figure S14, Supporting Information).

Quantification of the uptake of GLuc‐Cy5/PSomes‐RhoB into iPSC‐CMs was determined by flow cytometry, which revealed more than 95% of iPSC‐CMs were double positive for Cy5 and RhoB after 24 h of incubation, confirming that both cargo (GLuc) and carrier (PSomes) were associated with the iPSC‐CMs (Figure 3B and Figure S15, Supporting Information). As a control, the uptake of free GLuc‐Cy5 resulted in >90% positive iPSC‐CMs, although a comparison of the mean fluorescence intensity revealed significantly higher uptake of GLuc/PSomes which was attributed to the role of the PSomes in facilitating uptake through the cell membrane (Figure S16, Supporting Information). Further information regarding the uptake mechanism of these PSomes into iPSC‐CMs was also studied by measuring changes in cell uptake under selective inhibitory conditions (Figure S11B, Supporting Information). Performing uptake of GLuc‐Cy5/PSomes at 4 °C resulted in significant uptake inhibition of about 51 ± 8% according to flow cytometry, suggesting that endocytosis of these PSomes occurs via an energy‐dependent mechanism. This was further probed by pre‐incubating iPSC‐CMs for 1 h with three separate endocytosis pathway‐specific inhibitors.^[^ ^28^ ^]^ Filipin III, an inhibitor of caveolae‐mediated endocytosis, resulted in nonsignificant changes in uptake compared to the control, suggesting minimal involvement of caveolae in PSome uptake into iPSC‐CMs. In contrast, both chlorpromazine (CPZ) and 5‐(*N*‐ethyl‐*N*‐isopropyl)amiloride (EIPA), which have been employed to block the clathrin‐mediated pathway and macropinocytosis, respectively, significantly impeded endocytosis of PSomes by 34 ± 6% and 30 ± 5%. These results provide strong evidence for the role of both the clathrin‐mediated pathway and macropinocytosis in the cellular uptake mechanism of GLuc/PSomes into iPSC‐CMs.

Finally, to determine whether uptaken PSomes were significantly retained by the endolysosomal system, live confocal microscopy was performed 2 and 24 h post‐incubation with GLuc‐Cy5/PSomes, and acidic organelles were stained using LysoTracker Green (Figure S17, Supporting Information). Under these conditions, poor colocalization of GLuc‐Cy5/PSomes with the LysoTracker signal was observed at both time points, suggesting that the PSomes employed in this work can escape from the endolysosomal system; similar reports of endolysosomal escape of neutral PSome systems have previously been reported.^[^ ^2^, ^29^ ^]^ Taken together, these imaging results indicate that uptaken AOs do not colocalize significantly with either the nucleus, cell membrane (Figures S13 and S14, Supporting Information), or endolysosomal system (Figure S17, Supporting Information). In addition, since these AOs are PEGylated, specific targeting moieties are not presented on the particle surface and we therefore believe it likely that our AOs localize primarily within the cytoplasm of the iPSC‐CMs.

Encouraged by the successful uptake of GLuc/PSomes into iPSC‐CMs, we sought to determine whether the GLuc/PSomes could function as AOs to produce a bioorthogonal product, light, within the intracellular environment. This was achieved by first incubating iPSC‐CMs with GLuc or GLuc/PSomes for 24 h, to promote uptake, before washing the cells with fresh medium to remove non‐uptaken GLuc or GLuc/PSomes. Notably, GLuc/PSomes or GLuc that were internalized within iPSC‐CMs retained their ability to produce bioluminescence when the cells were treated with CTZ (Figure 3C). Although naturally produced GLuc is a secretory protein with some sensitivity to its surrounding environment (e.g., pH and ionic strength^[^ ^30^ ^]^), this maintenance of activity in the intracellular environment is consistent with intracellularly retained GLuc variants reported in the literature which have been developed as quantitative reporters.^[^ ^31^ ^]^ The luminescence activity of the uptaken GLuc/PSomes was also visualized on a cellular level using luminescence microscopy, confirming the potential of this AO system to produce light within the intracellular environment (Figure 3D and Figure S18 and Movie S2, Supporting Information).

Since synthetic AOs lack the ability to be continuously produced within cells, a system with long‐term intracellular stability is highly desirable for providing prolonged therapeutic action. To monitor the potential for long‐term AO activity, iPSC‐CMs were incubated with GLuc‐Cy5/PSomes‐RhoB for 24 h, washed with medium, and then cultured for a further 7 days with medium changes every other day. Flow cytometry indicated that after 7 days in culture, more than 90% of iPSC‐CMs remained double positive for Cy5 and RhoB (Figure 3E and Figure S15C, Supporting Information), while confocal microscopy confirmed continued intracellular colocalization of the protein (Cy5) and polymersome (RhoB) signals (Figures S13B and S19, Supporting Information). To directly determine the retention of enzymatic activity of the GLuc/PSomes after long‐term incubation, we measured the intracellular bioluminescence after maintaining the cells in culture for 3 and 7 days after GLuc/PSome internalization. Remarkably, GLuc/PSomes were capable of retaining 42.3 ± 8.3% of their original activity after 7 days inside the iPSC‐CMs, whereas under the same conditions, internalized free GLuc lost nearly all its luminescence activity (3.6 ± 0.4% activity retained) (Figure 3F). This agreed with the stability data obtained for GLuc/PSomes (Figure 2D,E), suggesting that the polymersome membrane could shield the encapsulated enzymes, even in the intracellular environment, leading to long‐term retention of enzymatic activity. Supporting the long‐term stability of uptaken GLuc/PSomes is the polymersome's resistance to biodegradation since its constituent polymers, PEG and the methacrylate‐based PHPMA, are generally considered nonbiodegradable in the literature.^[^ ^32^ ^]^ Taken together, the cellular uptake and long‐term stability of the intracellular bioluminescence of GLuc/PSomes demonstrate that PISA‐derived PSomes can deliver encapsulated enzymes into cells while still maintaining enzyme activity, all of which are key properties of AO systems.

**Figure 4:**
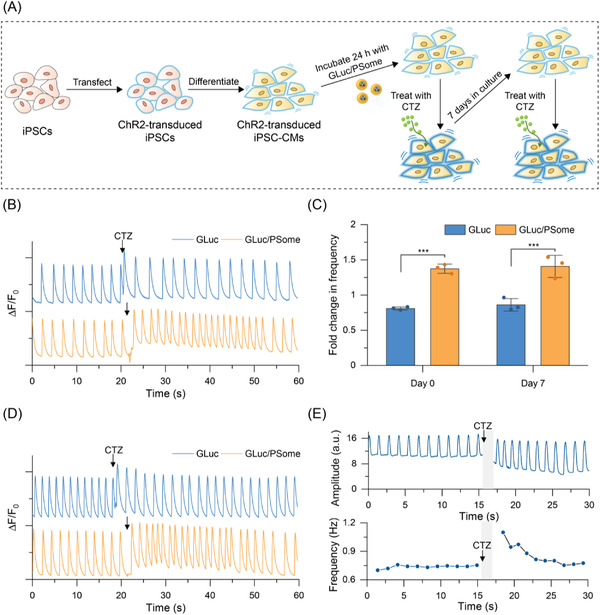
Ability of GLuc/PSomes to act as AOs for modulating optogenetic iPSC‐CMs. A) Schematic illustration showing the process to obtain genetically modified ChR2‐transduced iPSC‐CMs and their response to CTZ after taking up GLuc/PSomes as AOs. B,D) Recordings of calcium transients during CTZ treatment in ChR2‐transduced iPSC‐CMs with internalized GLuc/PSome or GLuc. Cells were incubated for 24 h with GLuc/PSome or free GLuc before B) treatment with CTZ (day 0), then cultured for an additional 7 days and D) re‐stimulated with CTZ (day 7). Cells were stained with Rhod‐4 AM prior to fluorescence imaging with all analysis calculated on the basis of the total image intensity of the entire field of view. CTZ addition (black arrow) was performed manually after ≈20 s to record sufficient baseline cardiomyocyte activity. C) Fold‐change in beating frequency of ChR2‐transduced iPSC‐CMs with internalized GLuc/PSome or GLuc after treatment with CTZ either immediately after incubation (day 0) or after an additional 7 days in culture (day 7). Beating frequencies were obtained by averaging the frequency of seven beats before and after addition of CTZ (mean ± SD, *n* = 3, ***p < 0.001 based on one‐way ANOVA with Tukey's multiple comparisons test). E) Change in mechanical beating amplitude and frequency of ChR2‐transduced iPSC‐CMs with internalized GLuc/PSome in response to CTZ. Images were analyzed using the “MYOCYTER” plugin for ImageJ. The gray bars represent the period whereby CTZ is directly added, resulting in imaging perturbations for ≈1.5 s.

The robust nature of the intracellular bioluminescence produced from GLuc/PSomes as AOs has significant potential to mediate a broad range of light‐sensitive chemical/biological processes within cells.^[^ ^33^ ^]^ For example, as an alternative to conventional external light sources, bioluminescence can be harnessed to trigger light‐sensitive ion channels by transduction of opsin‐luciferase fusion proteins into the membranes of neurons, as an excitable cell type.^[^ ^34^ ^]^ We hypothesized that light‐sensitive, genetically engineered iPSC‐CMs could function as an ideal cellular model to test the ability of our AOs to modulate cellular behaviors via AO‐produced bioluminescence. Such an approach would thereby demonstrate the potential for synergistic coupling of genetic engineering with synthetic AO implants leading to the creation of unique triggerable biohybrid cellular systems. First, iPSCs were transduced via lentiviral infection with ChR2, a light gated cation channel, fused to enhanced yellow fluorescent protein (EYFP) as a reporter protein, and subsequently differentiated into cardiomyocytes by a chemically defined differentiation protocol (**Figure** **4**A).^[^ ^35^ ^]^ The ChR2‐transduced iPSC‐CMs demonstrated spontaneous beating from day 7, with confocal microscopy confirming the presence of membrane‐bound EYFP reporter protein (Figure S20A, Supporting Information), and hence, the successful transduction of ChR2. The contraction of the iPSC‐CMs was monitored by recording the time course of calcium transients using an intracellular calcium‐sensitive dye, Rhod‐4 acetoxymethyl ester (Rhod‐4 AM). The light sensitivity of the ChR2‐transduced iPSC‐CMs was confirmed by irradiating the cells with pulsed 470 nm light‐emitting diode (LED) light at 1 Hz (pulse width: 100 ms) which led to light‐synchronized intracellular calcium spikes and associated cellular contraction (Figure S20B, Supporting Information). Alternatively, continuous 470 nm LED stimulation at constant intensity also induced an increase in the spontaneous beating rate, consistent with reports elsewhere.^[^ ^36^ ^]^

Having confirmed the light‐sensitive nature of ChR2‐transduced iPSC‐CMs, we sought to test our hypothesis that our light‐generating intracellular AOs could stimulate the beating of these optogenetically engineered iPSC‐CMs. We first confirmed the comparable uptake behavior of GLuc/PSomes into ChR2‐transduced iPSC‐CMs (Figure S14, Supporting Information) compared to wild‐type iPSC‐CMs as described above (Figure 3A and Figure S13, Supporting Information). To demonstrate the AO potential of GLuc/PSomes, ChR2‐transduced iPSC‐CMs were incubated with GLuc/PSomes for 24 h, washed with fresh medium to remove extracellular PSomes, and stained with Rhod‐4 AM to monitor calcium transients as a proxy for cardiac beating (Figure 4A). Upon addition of CTZ, a near instantaneous increase in the beating rate was observed for at least 10 s (Figure 4B, additional experimental replicates in Figure S21A,B, Supporting Information). Importantly, this demonstrated relatively rapid diffusional uptake of CTZ to the enzyme site (on the order of seconds), despite the presence of diffusional barriers (e.g., cell and polymersome membranes) and appears consistent with the relatively rapid luminescence kinetics that were observed intracellularly (Figure 3C). Interestingly, this relatively rapid biological effect has also been observed in GLuc/ChR2‐transfected neuronal cell models with stimulation typically being observed seconds after CTZ addition.^[^ ^34a^ ^]^

To quantify this effect, the frequencies of seven beats after adding CTZ were averaged and normalized to the average frequency of seven beats of spontaneous beating prior to CTZ addition. Based on this analysis, ChR2‐transduced iPSC‐CMs treated with GLuc/PSomes showed a statistically significant increase of the beating frequency (37 ± 7%, *n* = 3) upon CTZ addition compared to cells treated with free GLuc (*p* < 0.001) (Figure 4C). Indeed, ChR2‐transduced iPSC‐CMs treated with only free GLuc underwent a slight decrease in beating frequency upon addition of CTZ which was attributed to the well‐known sensitivity of the cardiomyocyte beating rate to the extracellular environment.^[^ ^37^ ^]^ This hypothesis was supported by similar slight decreases in beating rate upon CTZ treatment of ChR2‐transduced iPSC‐CMs without any internalized GLuc or wild‐type iPSC‐CMs with previously internalized GLuc/PSomes (Figure S22, Supporting Information). Two main reasons can be rationalized as to the inability of GLuc (as opposed to GLuc/PSomes) to stimulate beating of iPSC‐CMs via a luminescent‐optogenetic mechanism. First, flow cytometry data indicated significantly lower cellular uptake of free GLuc compared to GLuc/PSomes (adjusted to the same GLuc concentration added) which was attributed to the role of the PSome nanocarrier in facilitating uptake through the cell membrane (Figure S16B, Supporting Information). Second, free GLuc is highly susceptible to intracellular stresses (particularly proteases) resulting in a lower efficiency of luminescence production after cellular uptake compared to GLuc/PSomes (Figure 2D, 2E and 3F). Such a result, therefore, demonstrates the importance of the developed polymersome system in promoting stabilization and uptake of GLuc and ultimately enabling the pathway for stimulation of cardiomyocyte beating in response to CTZ treatment.

Having confirmed the ability of internalized GLuc/PSome to act as AOs to trigger optogenetic responses, we were interested to exploit their long‐term intracellular activity (Figure 3F) to study the persistence of their in vitro activity. To demonstrate the long‐term feasibility of this AO system, GLuc/PSomes internalized within ChR2‐transduced iPSC‐CMs that had been previously stimulated with CTZ (Figure 4B and Figure S21A,B, Supporting Information) were maintained in culture for an additional 7 days, before re‐staining with Rhod‐4 AM and restimulation with fresh CTZ (Figure 4A). Even after 7 days in culture, the bioluminescence generated from internalized GLuc/PSomes (following the addition of CTZ) was sufficient to trigger a statistically significant increase of the beating frequency (41 ± 16%) compared to cells previously treated with only free GLuc (*p* < 0.001) (Figure 4C,D) (additional experimental replicates in Figure S21C,D, Supporting Information). This demonstrated that GLuc/PSomes exhibited excellent intracellular stability over a period of at least 7 days in culture as well as the capability to be reactivated multiple times. Occasionally, calcium transient imaging indicated the occurrence of slight, temporary increases in the baseline intracellular calcium level upon the addition of CTZ (Figure 4B,D and Figure S21, Supporting Information). It should be noted that similar (albeit more abrupt and sustained) baseline drifts are commonly observed in more conventional optogenetic systems when applying constant stimulation from traditional light sources such as LEDs (Figure S20B, Supporting Information); in our system, this behavior is relatively minor owing to the distinct kinetic decay of bioluminescence as a light source. Overall, these results demonstrate the significant potential of GLuc/PSomes as AOs by showing that they can be readily internalized into iPSC‐CMs, remain inside cells for at least 7 days while retaining their catalytic activity, and modulate cellular behavior in a long‐term manner.

We further investigated the uniformity of the calcium transients over multiple cell regions by also analyzing multiple regions of interest (ROIs) across the imaging field of view (Figure S23, Supporting Information) and comparing them to the overall image intensity which was used to estimate the beating frequency changes (Figure 4B,D). Using this analysis, the individual ROIs showed highly synchronized calcium transients over the whole imaging field and this synchronized beating response was maintained upon treatment with CTZ for both the day 0 (Figure S23A,B, Supporting Information) and day 7 (Figure S23C,D, Supporting Information) stimulations. We also conducted preliminary investigation into the propagation wavefronts by generating activation maps (Figure S24, Supporting Information).^[^ ^38^ ^]^ Although some changes were seen in the propagation wave shape and direction in response to CTZ addition, there was no obvious evidence for adverse electrophysiological changes to the iPSC‐CMs induced by the CTZ stimulation. To further validate the biocompatibility of the GLuc/PSomes AOs, we also analyzed beating frequency and conduction velocity changes and observed no significant changes in the intrinsic beating behaviors of iPSC‐CMs for 7 days, such as changes in the pacing rate (Figure S25C, Supporting Information) or conduction velocities (Figure S25D, Supporting Information). As a proof‐of‐concept study, we have conducted this preliminary analysis using 2D monolayer cultures, but it should be noted that more insight could potentially be gained in future studies using a suitable 3D tissue construct (e.g., cardiac slices).

Beyond cyclic intracellular calcium changes, cardiomyocyte membrane depolarization is intrinsically linked to downstream mechanical beating due to excitation–contraction coupling. To highlight the ability of our combined AO and genetic engineering approach to produce cellular‐driven mechanical changes, we monitored the amplitude of iPSC‐CM beating under continuous brightfield imaging. As for calcium transient imaging, ChR2‐transduced iPSC‐CMs were incubated with GLuc/PSome AOs for 24 h and washed with fresh medium to remove extracellular PSomes. To monitor mechanical changes, the real‐time response to CTZ stimulation was examined under brightfield microscopy (Movie S3, Supporting Information). Analysis of the image frames was performed using the “MYOCYTER” ImageJ plug‐in which enables frame‐by‐frame tracking of the amplitude of iPSC‐CM beating.^[^ ^39^ ^]^ Using this analysis, an acceleration of the mechanical beating rate was observed for at least 5 s after CTZ addition (Figure 4E), confirming that the CTZ‐induced changes in calcium transient imaging (Figure 4B) were mirrored in the mechanical contraction behavior of the ChR2‐transduced iPSC‐CMs. This chemically induced optogenetic modulation by GLuc/PSome AOs demonstrated the ability of this system to mediate light‐responsive biological processes, and has the potential to be expanded to a range of applications, such as photosynthetic processes or even in biohybrid soft robotics by harnessing the inherent excitation‐coupled mechanical contraction of cardiomyocytes.

## 3 Conclusion

In this work, we have presented a downscaled, microliter volume (10 µL) synthesis of enzyme‐encapsulated PSomes based on the PISA approach. Reducing the scale of these syntheses (conventionally on the milliliter scale) opens up opportunities for the encapsulation of a broader range of otherwise cost‐prohibitive enzymes compared to conventional self‐assembly approaches such as solvent exchange or thin‐film rehydration. Such an advance is significant for broadening the biochemical mechanisms that can be modulated using an AO approach as well as facilitating higher throughput screening approaches. This microliter volume AO synthesis was leveraged to demonstrate the encapsulation of GLuc as a relatively expensive enzyme and one which possesses activity that can be conveniently monitored intracellularly via luminescent turnover of CTZ. We showed that PSome encapsulation endowed GLuc with prolonged luminescence kinetics and much greater resistance to thermal, proteolytic, and intracellular stresses compared to the free enzyme. We exploited this property to apply GLuc/PSomes as intracellular AOs, whereby CTZ‐induced bioluminescence could be harnessed to stimulate the beating of optogenetically modified iPSC‐CMs even after 7 days in the intracellular environment. We also demonstrated that this unique system could modulate the mechanical beating of optogenetically engineered iPSC‐CMs. To the best of our knowledge, this is the first demonstration of an AO system capable of selectively producing a cell‐mediated mechanical response. The generated AOs enable repeated modulation of cellular behavior even a week after AO uptake, highlighting the significant potential of this approach to generate long‐acting, highly robust, triggerable biohybrid cellular systems. This work therefore provides exciting proof‐of‐concept toward the broad opportunities that can be offered in synthetic biology by introducing aspects of synthetic materials chemistry such as the high stability and chemical flexibility of synthetic membrane constructs (e.g., polymersomes), to the existing genetic modification approaches applied in synthetic biology (e.g., optogenetics). Given the highly versatile nature of this platform, we expect that this work will significantly open up the field of AOs to novel and innovative applications across nanomedicine and bioengineering.

## 4 Statistical Analyses

Data were processed using Origin Pro 2020b. Where applicable, data has been represented as mean ± standard deviation with at least three replicates for each analysis. For Figure 4C, a one‐way analysis of variance (ANOVA) with Tukey's multiple comparisons test was employed to analyze the changes in beating frequency. The specifics associated with pre‐processing of data, sample sizes, and statistical methods, including post‐hoc test methods, are described further within the figure captions.

## 5 Ethical Approval

The iPSC line (WTC‐11) was a kind gift from Professor Bruce Conklin, The J. David Gladstone Institutes, USA. WTC‐11 was generated from a healthy male donor who signed a consent form. The protocol was approved by the UCSF Committee on Human Research, San Francisco, USA (study number 10–02521, “Induced Pluripotent Stem Cells for Genetic Research”).

## Conflict of Interest

The authors declare no conflict of interest.

## Author Contributions

H.K. and J.Y. contributed equally to this work. H.K. and J.Y. conceived the project concept and designed the experiments. H.K. performed all in vitro work and luminescence experiments. J.Y. prepared and characterized the polymersomes. A.N. performed the FCS measurements and analysis. H.K. and W.K.‐A. prepared the transfected optogenetic cell line. R.W. assisted with optogenetic measurements and analysis. O.R.‐G. and C.T. assisted with nanoparticle characterization. H.K. and J.Y. wrote the manuscript with feedback from all authors. M.M.S. supervised the project.

## Supporting information

Supporting Information

Supplemental Movie 1

Supplemental Movie 2

Supplemental Movie 3

## Data Availability

The data that support the findings of this study are available from rdm‐enquiries@imperial.ac.uk upon reasonable request.
