# Supplementary material for: Microliter Scale Synthesis of Luciferase‐Encapsulated Polymersomes as Artificial Organelles for Optogenetic Modulation of Cardiomyocyte Beating
Source: Adv Sci (Weinh). 2022 Jul 28;9(27):2200239. doi: 10.1002/advs.202200239 (PMC9507352; doi:10.1002/advs.202200239)
Supplement: Supplementary file 1 — Supporting Information [file ADVS-9-2200239-s001.pdf]

ADVANCED SCIENCE
Open Access

Supporting Information

for *Adv. Sci.*, DOI 10.1002/advs.202200239

Microliter Scale Synthesis of Luciferase-Encapsulated Polymersomes as Artificial Organelles for Optogenetic Modulation of Cardiomyocyte Beating

*Hyemin Kim, Jonathan Yeow, Adrian Najer, Worrapong Kit-Anan, Richard Wang, Omar Rifaie-Graham, Chalaisorn Thanapongpibul and Molly M. Stevens**

Supporting Information

Microliter scale synthesis of luciferase-encapsulated polymersomes as artificial organelles for optogenetic modulation of cardiomyocyte beating

*Hyemin Kim,† Jonathan Yeow,† Adrian Najer, Worrapong Kit-Anan, Richard Wang, Omar Rifaie-Graham, Chalaisorn Thanapongpibul, and Molly M. Stevens**

Dr. H. Kim, Dr. J. Yeow, Dr. A. Najer, Dr. W. Kit-Anan, Dr. R. Wang, Dr. O. Rifaie-Graham, C. Thanapongpibul, Prof. M. M. Stevens
Department of Materials, Department of Bioengineering, and Institute of Biomedical Engineering
Imperial College London
London SW7 2AZ, UK.
E-mail: m.stevens@imperial.ac.uk

† Authors contributed equally

Experimental Section

Materials

Poly(ethylene glycol) monomethyl ether (PEG_{113}, M_n = 5000 g mol^{-1}, Sigma-Aldrich), 4-cyano-4-[(dodecylsulfanylthiocarbonyl)sulfanyl] pentanoic acid (CDTPA, Boron Molecular), N,N'-dicyclohexylcarbodiimide (DCC, Sigma-Aldrich), 4-(dimethylamino)pyridine (DMAP, Sigma-Aldrich), sodium azide (Sigma-Aldrich), glycidyl methacrylate (Sigma-Aldrich), methacryloxyethyl thiocarbamoyl rhodamine B (RhoBMA, Polysciences), sodium trimethylsilylpropanesulfonate (DSS, Sigma-Aldrich), mineral oil (BioReagent, Sigma-Aldrich), and all other reagents were used as received unless otherwise specified. Hydroxypropyl methacrylate (HPMA, Sigma-Aldrich) was purified by silica column chromatography (to remove crosslinking impurities) using ethyl acetate/hexane (1:8) as eluent prior to storage at -20 °C. Stock solutions of Cyanine5 NHS ester (Cy5-NHS, 12 mM, Lumiprobe) and Alexa Fluor® 488-DBCO (AF488-DBCO, 10 mM, Jena Bioscience) were prepared in DMSO and stored at -20 °C prior to use. *Gaussia* luciferase protein (GLuc) and coelenterazine (CTZ) native were purchased from Nanolight Technology (Pinetop, AZ). GLuc was resuspended in DPBS (2 mg mL^{-1}), aliquoted, and stored at -80 °C prior to use. CTZ was resuspended in propylene glycol (10 mM), stored at -80 °C, diluted in aqueous buffer or medium to the desired concentration immediately prior to use. Sodium dodecyl sulfate (SDS), elastase from porcine pancreas, rhodamine B (RhoB) isothiocyanate, sodium lactate solution, HEPES-buffered saline, monoclonal anti-α-actinin antibody, Filipin III from Streptomyces filipinensis (Filipin), chlorpromazine hydrochloride (CPZ), and 5-(N-ethyl-N-

isopropyl)amiloride (EIPA) were purchased from Merck KGaA (Darmstadt, Germany). The induced pluripotent stem cell (iPSC) line (WTC-11) was a kind gift from Professor Bruce Conklin, Gladstone Institute, USA. WTC-11 was generated from a healthy male donor who signed a consent form approving the donation of iPSCs to the public stem cell bank of the Coriell Institute (catalog no. GM25256). The protocol for derivation and use of human iPSCs was approved by the UCSF Committee on Human Research, San Francisco, CA (study number 10-02521, "Induced Pluripotent Stem Cells for Genetic Research"). The plasmids, pLenti-EF1a-hChR2(H134R)-EYFP-WPRE, pCMVdeltaR8.74, and pMD2.G were kindly provided by Prof Karl Deisseroth of Stanford University. Micro BCA™ protein assay kit, illustra NAP-5™ columns, 293FT cell line, Essential 8™ medium, Dulbecco's modified essential medium (DMEM)/F-12 medium, DMEM medium with high glucose and GlutaMAX™, Opti-MEM™ reduced serum medium, RPMI 1640 medium, Dulbecco's PBS (DPBS), fetal bovine serum (FBS), B-27™ supplement minus insulin, B-27™ supplement, Collagenase type II, PrestoBlue™ Cell Viability Reagent, TrypLE™ Express Enzyme, Alexa Fluor™ 488-conjugated wheat germ agglutinin (WGA), Alexa Fluor™ 488-conjugated donkey anti-mouse IgG secondary antibody, LysoTracker™ Green DND-26, Hoechst 33342 solution, SlowFade™ Diamond Antifade Mountant, and HEPES-buffered Tyrode's solution were obtained from Thermo Fisher Scientific (Waltham, MA). Matrigel® Growth Factor Reduced Basement Membrane Matrix was purchased from Corning (Corning, NY). Y-27632 was obtained from Stemcell Technologies (Cambridge, UK). CHIR99021 was purchased from Tebubio (Yvelines, France). Wnt-C59 was purchased from Stratech Scientific (Ely, UK). Chambered coverslips with 8 wells were purchased from ibidi (Gräfelfing, Germany). CalciFluor™ Rhod-4 acetoxymethyl ester (Rhod-4 AM) was purchased from Santa Cruz Biotechnology (Dallas, TX). Alexa Fluor® 647 mouse anti-cardiac troponin T (cTnT) was obtained from BD Biosciences (Franklin Lakes, NJ)

Instrumentation

All photopolymerization reactions were performed using a Teleopto LAD-1 LED array driver powering a LEDA-V (λ_{max} = 405 nm) LED array. Light intensity was measured using a Thorlabs PM100D power meter equipped with a S121C photodiode. Unless otherwise stated, all photopolymerizations were conducted in a flat bottom, HiBase, clear 1536-well polystyrene microplate (Greiner Bio-One, 782101).

Unless otherwise stated, all ^{1}H-NMR spectra were recorded in DMSO-d6 (VWR, ≥ 99.9%) using a JEOL 400 MHz spectrometer. All chemical shifts are reported in ppm (δ) relative to tetramethylsilane, referenced to the chemical shifts of residual solvent resonances.

Polymer molecular weight ($M_{n,GPC}$) and dispersity (*Đ*) were measured using a 1260 Infinity II GPC MDS (refractive index detection only) equipped with a PSS GRAM guard column (8 x 50 mm, 10 µm) and two PSS GRAM linear columns (8 x 300 mm, 10 µm, 500-1 000 000 Da) The eluent was HPLC grade DMF containing 0.075% (w/w) LiBr and running at a flow rate of 1 mL min^{-1} at 40 °C. Molecular weight calibration was performed using near-monodisperse poly(methyl methacrylate) standards (EasiVial, Agilent).

Dynamic light scattering (DLS) measurements were performed using a ZetaSizer Nano ZS at a scattering angle of 173°. All nanoparticle dispersions were measured in DPBS using disposable polystyrene microcuvettes.

For cryo-EM, 3.0 µL of nanoparticle dispersions in DPBS were applied to either glow-discharged Quantifoil R2/2 holey carbon grids (Quantifoil Micro Tools GmbH, Großlöbichau, Germany) or 400 mesh Cu-grids (TAAB Laboratories Equipment Ltd, Aldermaston, England) covered with an additional thin continuous carbon film. Frozen-hydrated specimens were prepared with an automatic plunge freezer FEI Vitrobot (Thermo Fisher Scientific, Waltham, MA, USA) operated at 16 °C and 100% relative humidity. The samples were incubated for 10 s on the grids, blotted for 3 to 4 s and plunged into liquid ethane. The cryo-specimens were transferred to a JEOL JEM-2100F transmission electron microscope (JEOL Ltd., Tokyo, Japan) operated at 200 kV. Images were recorded using TVIPS TemCam-XF416 CMOS camera (Tietz Video and Image Processing Systems GmbH, Gauting, Germany).

Luminescence output spectra were obtained using a Fluorolog®-3 (Horiba) spectrophotometer with an integration time of 10 ms, exit slit width of 29.4 nm and with dark offset correction.

A commercial LSM 880 (Carl Zeiss, Jena, Germany) was employed for FCS and FCCS data acquisition. All the data were fitted using PyCorrfit program 1.1.6.[1] The x-y dimension of the confocal volume (ω_{xy}^2) was calibrated using solutions of OG488 or Alexa647 in PBS. This allowed calculation of the diffusion coefficients (D) of the experimental samples by using the obtained diffusion times (τ_D) from the autocorrelation fits:

$$D = \frac{\omega_{xy}^2}{4\tau_D}$$

All measurements were performed at 37 °C, hence, a correction for the higher temperature was included for the diffusion coefficients: OG488 (D = 5.49 x 10^{-6} cm^2 s^{-1} at 37 °C, D = 4.1 x 10^{-6} cm^2 s^{-1} at 25 °C) and Alexa647 in PBS (D = 4.42 x 10^{-6} cm^2 s^{-1} at 37 °C, D = 3.3 x 10^{-6} cm^2 s^{-1} at 25 °C).[2] An Ar^+ laser (488 nm excitation) and HeNe-laser (633 nm excitation) and appropriate filter sets were used to obtain the fluorescence intensity fluctuations for single (FCS) or dual channel configuration (FCCS). Measurements were conducted 200 µm above the glass surface of the sample chamber (80827, ibidi, Germany) and using a 40x C-Apochromat water immersion objective (NA 1.2). Sample volumes were 5 µL. For each sample, either 25 x 5 s intensity traces were recorded for fast diffusing species (dyes, labeled enzymes) or 25 x 10 s for slow-diffusing polymersome samples. Intensity traces were then automatically auto-, and cross-correlated. These raw curves are always shown as the average curves across the entire measurement time of 150 s and 300 s, respectively. The following one component fits ($G_{1comp}(\tau)$) were used for curve fitting:

$$G_{1comp}(\tau) = \left(1 + \frac{T}{1-T} e^{\frac{-\tau}{\tau_{trip}}}\right) * \frac{1}{N * \left(1 + \frac{\tau}{\tau_D}\right) * \sqrt{1 + \frac{\tau}{SP^2 \tau_D}}}$$

τ_D is the diffusion time, τ_{trip} is the triplet time (fixed between 1 – 10 µs) of triplet fraction T; N is the effective average number of diffusing species within the confocal volume and SP is the structural parameter (fixed to 5). The Einstein-Stokes equation was then employed to calculate hydrodynamic radii (R_h) *via* the obtained diffusion coefficients (D). To obtain the number of cargo/label per polymersome, the counts per particle (cpp) for loaded polymersomes was divided by cpp for free dye or labeled GLuc. Since only 10% of loaded GLuc were labeled, the obtained values were multiplied by 10 to yield the final loading numbers.

For FCCS, a standard FCCS control sample was used to calibrate the maximum cross-correlation amplitude (FCCS Standard, IBA Sciences, 5-0000-504). The relative cross-correlation amplitude θ was calculated by:

$$\theta = \frac{G_{0,x}}{G_{0,green}}$$

where $G_{0,x}$ is the cross-correlation amplitude and $G_{0,green}$ is the autocorrelation amplitude of the green channel, both at τ = 0.[3]

Flow cytometry measurements were performed using a BD LSRFortessa™ Cell Analyzer (BD Biosciences, Franklin Lakes, NJ) with four lasers (Violet 405 nm, Blue 488 nm, Yellow-Green 561 nm and Red 640 nm). All samples were fixed with 4% (w/v) paraformaldehyde, washed, and finally resuspended in DPBS. Data were analyzed using Flowjo software (BD Biosciences).

Confocal imaging was performed using a Leica SP5 resonant inverted confocal microscope (Leica Microsystems, Germany) with a 63x oil immersion objective lens (Leica). Images were processed using ImageJ software (National Institutes of Health, Bethesda, MD).

Widefield imaging, including luminescence imaging, live-cell imaging, and calcium imaging, was performed using a Zeiss Axio Observer (Carl Zeiss, Oberkochen, Germany). Live cell imaging was carried out at 37°C with 5% CO_2 using a live cell environmental control system. Images were processed using ImageJ software.

Methods

Synthesis of PEG_{113}-CDTPA

The synthesis of PEG_{113}-CDTPA was performed using a protocol from literature.[4]

Synthesis of GLuc loaded PEG_{113}-b-PHPMA polymersomes (GLuc/PSomes) at 10 µL volumes

A typical synthesis for the preparation of GLuc loaded PEG-*b*-PHPMA polymersomes was as follows: PEG_{113}-CDTPA (11.38 µL of a 10 mg mL^{-1} acetone stock solution, 2.11×10^{-5} mmol) was added to a 1.5 mL low protein binding microcentrifuge tube and the organic solvent was allowed to evaporate. HPMA (1.22 mg, 1.14 µL, 8.46×10^{-3} mmol) was added, followed by 8.86 µL of GLuc (1 mg mL^{-1}) in DPBS. The tube was vortexed for ~30 s and 10 µL of this solution was transferred to a clear 1536-well microplate (Greiner Bio-One). To minimize evaporation during synthesis, 2.5 µL of mineral oil was gently pipetted on top of the polymerization solution. The microplate was then immediately irradiated from above using an LED array (λ_{max} = 405 nm, I ~ 10 mW cm^{-2}) for 3 h. To purify the polymersome samples, the turbid solution was removed from the well and 10 µL of DPBS was added to wash the well. The polymersomes were then diluted to a final volume of 100 µL with DPBS and spun at 16 000 × g for 10 min. The supernatant (containing residual monomer, unencapsulated GLuc, mineral oil as well as residual hydrophilic chains) was carefully removed and the polymersome pellet resuspended in 100 µL fresh DPBS. The centrifugation process was repeated an additional two times to obtain the purified PEG-*b*-PHPMA polymersomes. For calculation of

monomer conversion, DSS (final concentration ~3.8 mM) was added as a ^{1}H NMR internal standard. Conversions were determined in DMSO-d6 by monitoring the disappearance of the vinylic protons (δ 6.2 - 5.4 ppm) relative to the DSS peak at 0.0 ppm. GPC derived molecular weight values ($M_{n,GPC}$) and polymer dispersities ($Đ$) were determined against PMMA standards for both crude ($M_{n,GPC}$ = 69 100 ± 2 400 g mol^{-1}, $Đ$ = 1.52 ± 0.01) and purified ($M_{n,GPC}$ = 83 600 ± 3 800 g mol^{-1}, $Đ$ = 1.35 ± 0.01) GLuc-loaded polymersome samples (n = 3, synthetic replicates, mean ± SD). Control polymersomes synthesized in the absence of GLuc were also characterized by GPC before ($M_{n,GPC}$ = 68 200 ± 1 100 g mol^{-1}, $Đ$ = 1.54 ± 0.01) and after ($M_{n,GPC}$ = 80 200 ± 100 g mol^{-1}, $Đ$ = 1.36 ± 0.01) purification (n = 3, synthetic replicates, mean ± SD).

Upscaled synthesis of PEG_{113}-b-PHPMA polymersomes at 100 μL volume

Polymersomes were synthesized at a 100 μL reaction volume by following the above protocol but at a 10 times scale. To account for the greater reaction volume, photopolymerization was conducted in a clear 384-well microplate (Corning®).

Determination of encapsulation efficiency using μBCA

The loading efficiency of GLuc in GLuc/PSomes was calculated by quantifying the concentration of GLuc using a Micro BCA™ assay kit. The supernatants taken from each purification step and the final polymersome solution after purification were analyzed. In order to release the encapsulated GLuc for reaction with the assay reagents, the polymersomes were first lysed by adding 4% (w/v) SDS as a detergent, vortexing, and then spinning down the samples. All other samples, including the GLuc standard solutions (0.69 – 40 μg mL^{-1}, 1.5 fold serial dilutions) and supernatants, were also treated with 4% (w/v) SDS in the same manner. The samples (20 μL) were incubated with the premixed Micro BCA™ reagents (20 μL) in a low volume 384-well plate for 3 h at 37 °C and the absorbance at 540 nm was measured using a microplate reader (SpectraMax M5, Molecular Devices, San Jose, CA).

Luminescence measurements

50 μL of free GLuc (100 ng mL^{-1}) and GLuc/PSomes (adjusted to 100 ng mL^{-1} of encapsulated GLuc based on the Micro BCA™ assay) were loaded into each well of the white 96-well plate. Then, 50 μL of a freshly prepared 100 μM coelenterazine substrate solution was added to each well at a dispensing speed of 200 μL s^{-1} using an automatic dispenser to ensure accurate and immediate measurements of luminescence. Luminescence was measured at 1 s intervals using a plate reader (EnVision multimode plate reader, PerkinElmer, Waltham, MA). The maximum

luminescence after 5 s was used as a representative value to determine the relative activity changes of the GLuc in the stability test. Long-term luminescence kinetics were measured using the same method but on a SpectraMax M5 plate reader to account for the longer measurement time.

Repeated CTZ stimulation of GLuc/PSomes

50 µL of GLuc/PSomes (adjusted to 5 µg mL^{-1} of encapsulated GLuc based on the Micro BCA™ assay) was loaded into a single well of a white 96-well plate. Then, 50 µL of a freshly prepared 25 nM coelenterazine substrate solution was added at a dispensing speed of 200 µL s^{-1} using an automatic dispenser to ensure accurate and immediate measurements of luminescence. Luminescence was measured for 2 minutes at 1 s intervals using a plate reader (EnVision multimode plate reader, PerkinElmer, Waltham, MA). This CTZ addition was repeated an additional two times into the same well with 10 min intervals between luminescence measurements. Note that fresh CTZ solution must be prepared immediately before each measurement in order to minimize premature oxidation of CTZ under aqueous conditions.

Synthesis of azide functional methacrylate (AzHPMA)

AzHPMA was synthesized using a modified protocol from literature.[5] Briefly, sodium azide (1 g, 1.53×10^{-2} mol) and sodium bicarbonate (0.75 g, 8.93 mmol) were dispersed in 15 mL of a water/THF mixture (5:1) in a round bottom flask. The flask was subsequently sealed with a rubber septa and bubbled with nitrogen. To this suspension, glycidyl methacrylate (GMA, 1 mL, 7.33 mmol) was added dropwise with continuous nitrogen bubbling. When addition of GMA was complete, the flask was bubbled with nitrogen for an additional 5 min and then allowed to proceed at room temperature for 72 h in the dark under a static nitrogen atmosphere. The mixture was subsequently extracted with DCM (3 × 50 mL) and the combined organic extracts dried with magnesium sulfate. After filtration, the DCM was removed under a flow of compressed air, affording the mixed isomeric product (85/15), 3-azido-2-hydroxypropyl methacrylate (major) and 2-azido-3-hydroxypropylmethacrylate (minor) (AzHPMA) (0.404 g, 2.18 mmol, yield 30%). ^{1}H NMR (400 MHz, $CDCl_3$, δ): 6.18/6.15, 5.66/5.63 ($H_2C{=}C$); 4.07/5.07 (–CH–); 4.24/3.82 (–COOCH_2–); 3.43/3.55 (–CH_2X); 2.22 (–OH); 1.96/1.97 (–CH_3).

Labeling of GLuc with Cy5-NHS

To label GLuc with Cy5, a 10 fold molar excess of Cy5-NHS (12 mM in DMSO) was added to a solution of GLuc (1 mg mL^{-1}) in DPBS, pH 7.4 and the reaction was allowed to stir at room temperature in the dark. After 1 h, the labeled protein was purified using a NAP-5™ column using DPBS as eluent. The purified fraction was subsequently lyophilized and resuspended in water before use.

Synthesis of RhoBMA and GLuc-Cy5 dual labeled polymersomes for flow cytometry and confocal microscopy

PEG_{113}-CDTPA (11.38 µL of a 10 mg mL^{-1} acetone stock solution, 2.11×10^{-5} mmol) was added to a 1.5 mL low protein binding microcentrifuge tube and the organic solvent was allowed to evaporate. RhoBMA (7.02 µL of a 1 mg mL^{-1} acetone stock solution, 1.05×10^{-5} mmol) was subsequently added and the organic solvent was again allowed to evaporate. HPMA (1.22 mg, 1.14 µL, 8.46×10^{-3} mmol) was added, followed by 8.86 µL of GLuc-Cy5 (1 mg mL^{-1}) in DPBS. The tube was vortexed for ~30 s and 10 µL of this solution was transferred to a clear 1536-well microplate. To minimize evaporation during synthesis, 2.5 µL of mineral oil was gently pipetted on top of the polymerization solution. The microplate was then immediately irradiated from above using an LED array (λ_{max} = 405 nm, I ~ 10 mW cm^{-2}) for 3 h. The obtained polymersomes were subsequently purified as described above.

Synthesis of AF488 and GLuc-Cy5 dual labeled polymersomes (GLuc-Cy5/PSome-AF488) for FCCS

PEG_{113}-CDTPA (11.38 µL of a 10 mg mL^{-1} acetone stock solution, 2.11×10^{-5} mmol) was added to a 1.5 mL low protein binding microcentrifuge tube and the organic solvent was allowed to evaporate. AzHPMA (3.8 µL of a 1 mg mL^{-1} acetone stock solution, 2.11×10^{-5} mmol) was subsequently added and the organic solvent was again allowed to evaporate. HPMA (1.22 mg, 1.14 µL, 8.46×10^{-3} mmol) was added, followed by 8.86 µL of a mixture of GLuc-Cy5 and GLuc (overall [GLuc] = 1 mg mL^{-1}, 1:9 ratio) in DPBS. The tube was vortexed for ~30 s and 10 µL of this solution was transferred to a clear 1536-well microplate. To minimize evaporation during synthesis, 2.5 µL of mineral oil was gently pipetted on top of the polymerization solution. The microplate was then immediately irradiated from the top using an LED array (λ_{max} = 405 nm, I ~ 10 mW cm^{-2}) for 3 h. The obtained azide functional GLuc-Cy5 loaded polymersomes (GLuc-Cy5/PSomes) were subsequently purified as described above. To label the azide side chains of the polymer, the GLuc-Cy5/PSome pellet was resuspended in 100 µL DPBS. A 20 µL aliquot of the polymersome solution was further diluted to 100 µL in DPBS

before the addition of 2 µL of AF488-DBCO (10 µM in DMSO). The nanoparticle dispersion was vortexed for ~30 s before incubation at 37 °C overnight and in the dark. Finally, the dual labeled polymersome (GLuc-Cy5/PSome-AF488) was purified by three cycles of centrifugation (16 000 × g for 10 min) to remove unreacted dye followed by resuspension in DPBS.

Thermal stability of GLuc and GLuc/PSomes

1 mL of free GLuc (100 ng mL^{-1} in DPBS) and GLuc/PSomes (adjusted to 100 ng mL^{-1} of encapsulated GLuc in DPBS) were incubated at 37 °C and the luminescence was measured to monitor the enzyme activity. Aliquots were taken at pre-determined time points (1, 2, 3, and 4 weeks after starting the incubation) and stored at -80 °C. The luminescence of all samples (adjusted to an effective GLuc concentration of 100 ng mL^{-1}) was measured at the endpoint as described above. The maximum luminescence intensity of each sample at the starting point was set to 100% and other data were normalized to it.

Protease stability of GLuc and GLuc/PSomes

10 µL of free GLuc, GLuc/PSomes, and empty PSomes spiked with free GLuc (all adjusted to 1 µg mL^{-1} GLuc in DPBS) were incubated with 0.5 µg mL^{-1} elastase in a thermomixer (30 °C, 350 rpm) for 24 h to study the stability of GLuc against proteolysis. Samples incubated with the same conditions but without elastase were employed as controls. The luminescence of the samples (adjusted to an effective GLuc concentration of 100 ng mL^{-1}) was measured to monitor the enzyme activity at the endpoint as described above. The maximum luminescence intensity of the control samples, incubated without elastase, was set to 100% and other data were normalized to it.

iPSC culture

The iPSC line (WTC-11) was maintained in complete Essential 8™ medium on 6-well plates coated with Matrigel® diluted in DMEM/F12. The cells were regularly passaged every 3 days using 1:12 split ratio and the cells were cultured with 10 µM Y-27632 for the first 24 h after passaging to avoid cell dissociation-induced apoptosis.

Lentivirus production

Lentiviral particles were produced using 293FT cells as described elsewhere.[6] Briefly, 293FT cells were maintained in D10 medium (DMEM medium with high glucose and GlutaMAX™ supplemented with 10% (v/v) FBS and 1% (v/v) Penicillin-Streptomycin) and treated with the

mixture of the plasmids, pLenti-EF1a-hChR2(H134R)-EYFP-WPRE, pCMVdeltaR8.74, and pMD2.G, and 2M calcium chloride in HEPES-buffered saline overnight. The following day, the cells were washed with fresh D10 medium and incubated with Opti-MEM™ reduced serum medium with 1% (w/v) sodium pyruvate and 5 mM sodium butyrate for 24 h to produce lentiviral particles. The virus-containing supernatant was collected on the next day and filtered through a 0.45 μm filter flask. The filtered supernatant was concentrated using centrifugal filters (MWCO: 100kDa), aliquoted, and stored at -80 °C before use. The virus titer was determined by infecting 293T cells and analyzing the population of the transduced cells by flow cytometry.

Lentiviral infection of iPSCs

The iPSCs were seeded on a 48-well plate coated with Matrigel at a density of 2×10^4 cells per well and incubated for 24 h. The cells were infected by the lentiviral particles (multiplicity of infection = 2) with polybrene (8 μg mL^{-1}) in the complete medium for 24 h. The EYFP expression was monitored to confirm the transduction. The ChR2-transduced iPSC line was maintained using the same method as the precursor iPSCs.

Cardiac differentiation

Cardiac differentiation was optimized from a previously reported protocol.[7] Medium was changed to the differentiation medium, RPMI supplemented with 2% (v/v) B27-insulin supplement, when the cells had reached ~85% confluence. From day 0 to day 2, the differentiation medium was supplemented with 6 μM CHIR99021 and replaced with fresh differentiation medium on day 2. From day 3 to day 5, the differentiation medium was supplemented with 2.5 μM Wnt-C59. On day 5, this medium was replaced with fresh differentiation medium. Medium was then switched to RPMI supplemented with 2% (v/v) B27 supplement on day 7 and replaced with fresh medium every other day. Spontaneous contraction of the cells was observed from day 7. Medium was switched to RPMI without glucose, supplemented with 2% (v/v) B27 supplement and 5 mM sodium lactate, and changed every other day for the metabolic selection of cardiomyocytes from day 11 to day 17. After metabolic selection, the differentiated cardiomyocytes were dissociated by treating with collagenase type II for 3 h and replated to a Matrigel®-coated plate for further experiments. The cardiomyocytes were maintained in maintenance medium, which is RPMI supplemented with 2% (v/v) B27 supplement. To check the purity of cardiomyocytes, the cells were fixed with 1% (w/v) paraformaldehyde for 20 min, permeabilized with cold 90% (v/v) methanol for 10 min, stained

with 1:200 diluted Alexa Fluor® 647 mouse anti-cTnT in 0.5% (w/v) bovine serum albumin solution in DPBS with 0.1% (v/v) Triton for 1 h at room temperature, and then analyzed by flow cytometry. The same method was used for the ChR2-transduced iPSC line.

Cell viability test

The iPSC-derived cardiomyocytes (iPSC-CMs) were seeded on a Matrigel®-coated 96-well plate at a density of 1×10^5 cells per well and incubated for 24 h. The cells were treated with 100 μL of empty PSomes or GLuc/PSomes of the varied concentrations (the range of concentrations: 25 – 1600 μg mL^{-1}, serial two-fold dilutions) in the maintenance medium and incubated for 3 days at 37°C. The cell viability was evaluated by treating 100 μL of 1/10 diluted PrestoBlue™ Cell Viability Reagent for 4 h and measuring the fluorescence intensity (excitation: 560 nm, emission: 590 nm) using a plate reader (SpectraMax M5, Molecular Devices, San Jose, CA). Triplicate wells were used for each condition. The cell viability was normalized to the cells not treated with any samples.

Flow cytometry

The iPSC-CMs were seeded on a Matrigel®-coated 48-well plate at a density of 2×10^5 cells per well and incubated for 24 h. The cells were incubated with 200 μL of GLuc-Cy5 (200 ng mL^{-1}) or GLuc-Cy5/PSomes-RhoB (adjusted to 200 ng mL^{-1} of GLuc-Cy5) in the maintenance medium for 24 h. The cells were washed three times with DPBS and collected by treating with TrypLE™ Express Enzyme for 10 min. The cells were fixed by 4% (w/v) paraformaldehyde for 15 min, resuspended in DPBS, and filtered through a 40 μm mesh to remove aggregates. To monitor long-term behavior, the cells were cultured in the maintenance medium for a further 7 days after incubation and the samples were prepared using the same method. To study cellular uptake kinetics, cells were incubated with 200 μL of GLuc-Cy5/PSomes (adjusted to 200 ng mL^{-1} of GLuc-Cy5) in the maintenance medium for 1 h, 2 h, 4 h, and 24 h, and the cells were collected for the analysis. To determine the effect of temperature on uptake, cells were incubated with GLuc-Cy5/PSomes for 1 h at 4°C. To study uptake mechanisms, cells were pre-incubated with three endocytosis inhibitors, Filipin (1 μg mL^{-1}), CPZ (10 μg mL^{-1}), or EIPA (50 μM) for 1 h, and incubated with GLuc-Cy5/PSome for an additional 1 h at 37°C. The uptake under these inhibitory conditions was compared with the control (inhibitor-free uptake for 1 h at 37°C). Triplicate wells were used for each condition. All the measurements were carried out using a flow cytometer and the data were analyzed using FlowJo software.

Confocal microscopy

The iPSC-CMs were seeded on Matrigel®-coated chambered coverslips with 8 wells at a density of 2×10^5 cells per well and incubated for 24 h. The cells were incubated with 200 µL of GLuc-Cy5/PSomes-RhoB (adjusted to 200 ng mL^{-1} of GLuc-Cy5) in the maintenance medium for 24 h. The cells were washed with DPBS twice and fixed by 4% (w/v) paraformaldehyde for 15 min at room temperature. The cells were stained by either WGA to stain the cell membrane or anti-α-actinin antibody. For WGA staining, the cells were incubated with 1:500 diluted Alexa Fluor™ 488-conjugated WGA in DPBS for 15 min at room temperature. Alexa Fluor™ 488-conjugated WGA visualizes the cell membrane via binding to glycoproteins without the requirement for a permeabilization step using detergent. For the α-actinin staining, the cells were incubated with 0.2% (v/v) Triton-X-100 for 15 min for the permeabilization, the blocking solution for 1 h at room temperature, mouse anti-α-actinin antibody at 4°C overnight, and finally Alexa Fluor™ 488-conjugated donkey anti-mouse IgG secondary antibody for 1 h at room temperature. ChR2-EYFP-transduced iPSC-CMs were used for cell membrane stain-free imaging applying the same method. The nuclei of the cells were stained by treatment with DAPI for 3 min. The cells were mounted using antifade mountant. To monitor long-term behavior, the cells were cultured in the maintenance medium for a further 7 days after incubation and the samples were prepared using the same method. To monitor potential colocalization of PSomes with the endolysosomal system, cells were incubated with LysoTracker™ Green DND-26 for 1 h, followed by incubation of the cells with 200 µL of GLuc-Cy5/PSomes (adjusted to 200 ng mL^{-1} of GLuc-Cy5) in the maintenance medium for 2 h or 24 h. The cells were washed with DPBS twice and the nuclei of the cells were stained by treatment with Hoechst for 10 min. Imaging was performed using a confocal microscope and the images were processed using ImageJ software.

Live-cell microscopy

The iPSC-CMs were seeded on Matrigel®-coated chambered coverslips with 8 wells at a density of 2×10^5 cells per well and incubated for 24 h. The nuclei of the cells were pre-stained by treatment with Hoechst for 10 min. The cells were subsequently treated with 200 µL of GLuc-Cy5 /PSomes (adjusted to 200 ng mL^{-1} of GLuc-Cy5) in the maintenance medium and imaging was performed using a widefield microscope in a dark room. The cells were maintained at 37° with 5% CO_2 using a live cell environmental control system. The time-series fluorescence images were acquired at a sampling interval of 2 min for 24 h. The images were processed using ImageJ software.

Luminescence measurement of cells

The iPSC-CMs were seeded on a white/clear-bottom 96-well plate coated with Matrigel® at a density of 1×10^5 cells per well and incubated for 24 h. The cells were treated with 100 µL of GLuc (200 ng mL^{-1}) or GLuc/PSomes (adjusted to 200 ng mL^{-1} GLuc) in the maintenance medium for 24 h. After incubation, the cells were washed three times with maintenance medium. To monitor luminescence kinetics, the medium was replaced with 50 µL DPBS, the automatic dispenser of the plate reader was used to add 50 µL of 100 µM coelenterazine substrate solution each well and the luminescence was immediately measured at 1s intervals for 180 s. To monitor long-term behavior, the cells were cultured in the maintenance medium for a further 3 days or 7 days after incubation and the luminescence of all samples was measured at the endpoint. The maximum luminescence intensity of each sample at the starting point was set to 100% and other data were normalized to it.

Luminescence imaging

The iPSC-CMs were seeded on Matrigel®-coated confocal dishes at a density of 2×10^5 cells on the circular center with a diameter of 20 mm and incubated for 24 h. The cells were incubated with 200 µL of blank medium or GLuc/PSomes (adjusted to 200 ng mL^{-1} GLuc) in the maintenance medium for 24 h. The cells were washed three times with DPBS. Imaging was performed using a widefield microscope after removing emission filters in a dark room. The cells were treated with 1 mL of a freshly prepared 100 µM coelenterazine substrate solution and the time-series images were acquired at a sampling interval of 500 ms. The images were processed using ImageJ software.

Calcium imaging of ChR2-transduced iPSC-CMs stimulated by LED irradiation

The ChR2-transduced iPSC-CMs were seeded on Matrigel®-coated chambered coverslips with 8 wells at a density of 2×10^5 cells per well and incubated for 24 h. The cells were washed three times with DPBS and incubated with 200 µL of 10 µM Rhod-4 AM in HEPES-buffered Tyrode's solution for 20 min at 37 °C. The dye solution was removed from the cells and replaced with the fresh Tyrode's solution before imaging. Imaging was performed using a widefield microscope in a dark room and the time-series fluorescence images were acquired with a dichroic mirror and emission filter setting (excitation: 557 nm, emission: 572 nm) at a sampling interval of 10 ms for 60 s. The spontaneous contraction of the cells was monitored for the first 15 s, the light stimulation using a 470 nm LED array was turned ON for 30 s, and turned OFF for the last 15 s. Fluorescence intensity of each image frame over time was acquired using ImageJ software.

Long-term effect of PSomes on iPSC-CMs

The iPSC-CMs were seeded on Matrigel®-coated chambered coverslips with 8 wells at a density of 2×10^5 cells per well and incubated for 24 h. The cells were incubated with 200 μL of blank medium, or GLuc/PSomes (adjusted to 200 ng mL^{-1} GLuc) for 24 h. The cells were washed three times with DPBS and calcium imaging performed as described above. The spontaneous contraction of the cells was monitored for 30 s. After the experiment, the cells were washed three times with maintenance medium, cultured for a further 7 days, and the calcium imaging was repeated to monitor the long-term effect of GLuc/PSomes on the beating characteristics of iPSC-CMs. To calculate the beating frequency, the fluorescence intensity of each image frame was exported using ImageJ software and the time stamps of the peaks were obtained using MATLAB (MathWorks, Inc., Natick, MA, USA). The individual beating frequencies for each timepoint were calculated from the inverse of the time interval between adjacent peaks and the averaged beating frequency for 30 s was used for the analysis. Activation maps were obtained using a series of steps. A propagating wavefront was first identified in the recorded data, followed by a background subtraction to provide clear images of the wave dynamics. The images were then spatially binned, before the location of the propagating wave was identified as isochronal frames by performing a thresholding operation. Finally, the sequential isochronal frames were collapsed into a single frame such that earlier activation times overwrite latter activation times. The conduction velocity was calculated based on the activation map. The experiments were repeated in quadruplicate.

Bioluminescence-triggered optogenetic stimulation of cardiomyocytes

The ChR2-transduced-iPSC-CMs were seeded on Matrigel®-coated chambered coverslips with 8 wells at a density of 2×10^5 cells per well and incubated for 24 h. The cells were incubated with 200 μL of the medium, 200 ng mL^{-1} GLuc, or GLuc/PSomes (adjusted to 200 ng mL^{-1} GLuc) for 24 h. The cells were washed three times with DPBS and calcium imaging performed as described above. The spontaneous contraction of the cells was monitored for the first 20 s and the changes after adding 100 μL of a freshly prepared 1 μM coelenterazine substrate solution were observed for 40 s. After the experiment, the cells were washed three times with maintenance medium, cultured for a further 7 days, and the same stimulation experiment was repeated to monitor the long-term behavior.

For quantification, the total fluorescence intensity of each image frame was exported using ImageJ software and the time stamps of the peaks were obtained using MATLAB (MathWorks,

Inc., Natick, MA, USA). The individual beating frequencies for each timepoint were calculated from the inverse of the time interval between adjacent peaks. To quantify the effect of bioluminescence, the frequencies of seven beats before and after adding coelenterazine were averaged and compared. Activation maps were obtained as described above. The experiments were repeated in triplicate.

To analyze their mechanical contraction, the ChR2-transduced-iPSC-CMs were seeded on Matrigel®-coated 8-well chambered coverslips at a density of 2×10^5 cells per well and incubated for 24 h. The cells were incubated with 200 µL of GLuc/PSomes (adjusted to 200 ng mL^{-1} GLuc) in medium for 24 h. The cells were washed three times with DPBS and added with fresh Tyrode's solution.

Imaging was performed using a widefield microscope in a dark room with a brightfield imaging setup at a sampling interval of 10 ms for 60 s. The spontaneous contraction of the cells was monitored for the first 15 s before adding 100 µL of a freshly prepared 1 µM coelenterazine substrate solution with further observation for an additional 15 s. Analysis of the image frames was performed using the "MYOCYTER" ImageJ plug-in to estimate the amplitude changes of the movement over time.[8] The time stamps of the peaks were obtained using MATLAB (MathWorks, Inc., Natick, MA, USA) and beating frequency of the cells was calculated from the inverse of the time interval between adjacent peaks.

Statistical analysis

Data were processed using Origin Pro® 2020b. The specific pre-processing of data, sample sizes and statistical methods, including post-hoc test methods, are described in the figure captions.

Supplementary Figures

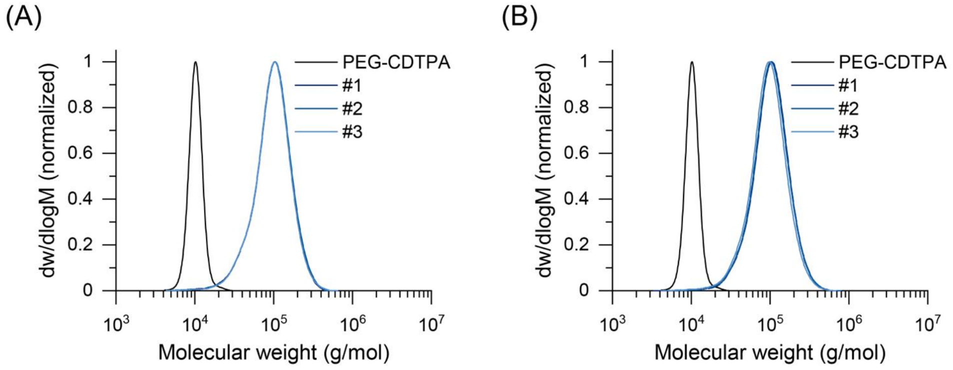

Figure S1. Molecular weight distributions of PEG-CDTPA and PEG-*b*-PHPMA block copolymers after purification by three cycles of centrifugation/resuspension. Polymersomes were synthesized in DPBS in the **(A)** absence or **(B)** presence of GLuc (n = 3, synthetic replicates).

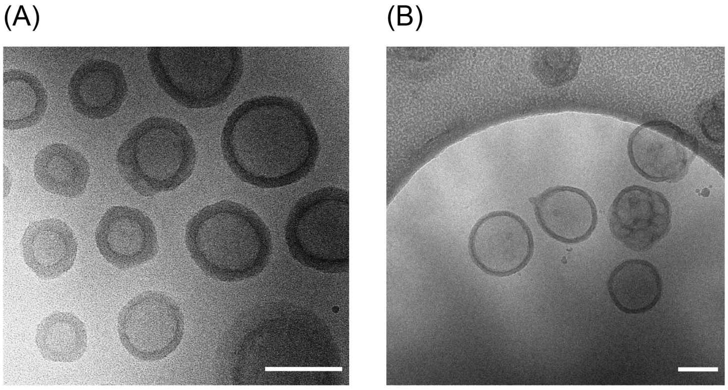

Figure S2. Additional cryoEM images of **(A)** GLuc/PSomes and **(B)** empty PSomes. Scale bar: 200 nm.

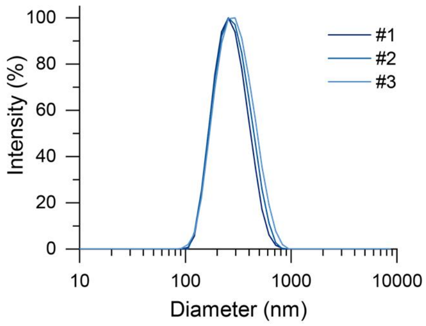

Figure S3. Average DLS intensity-based distribution of purified empty PSomes (data represent the mean of three repeated measurements for 3 synthetic replicates).

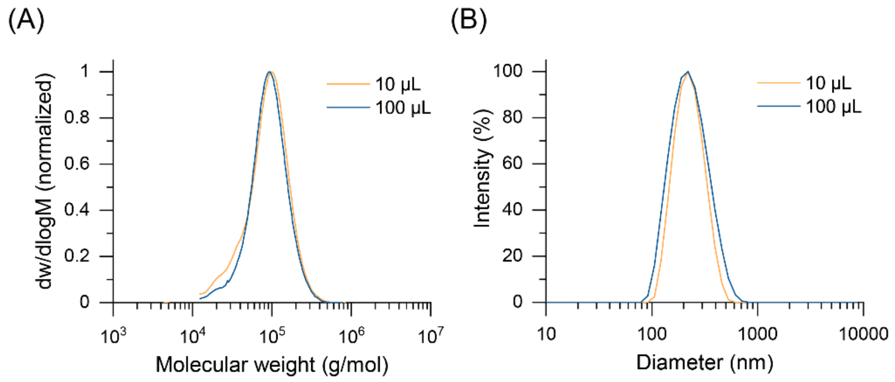

Figure S4. **(A)** Molecular weight distributions and **(B)** average DLS intensity-based distributions (data represent the mean of three repeated measurements) of purified PEG-*b*-PHPMA block copolymers synthesized at reaction scales of 10 µL (in a 1536-well plate) and 100 µL (in a 384-well plate).

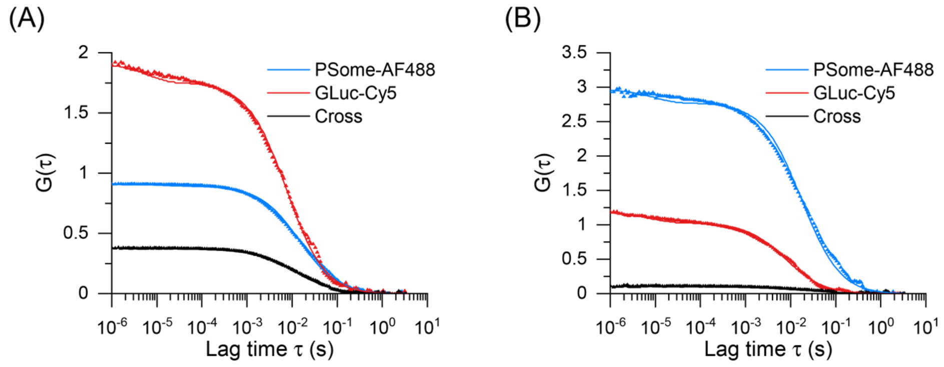

Figure S5. FCCS autocorrelation and cross-correlation curves for PSome-AF488 (488 nm excitation), GLuc-Cy5 (633 nm excitation), and cross channels for either **(A)** dual loaded GLuc-Cy5/PSome-AF488 or **(B)** physical mixtures of single labeled GLuc-Cy5/PSome and PSome-AF488 (average curves of $n = 25$ individual measurements).

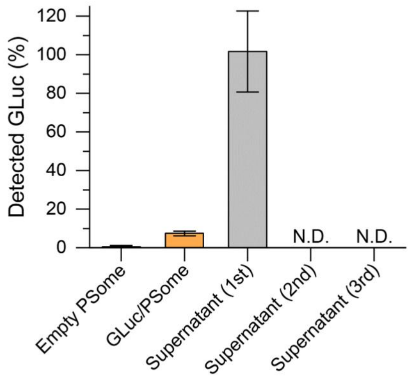

Figure S6. Quantification of GLuc in SDS-treated samples (GLuc/PSomes and empty PSomes after purification, and three supernatants obtained during the purification) calculated by Micro BCA™ assay. The amount of GLuc added for the synthesis was set to 100% (mean ± SD, n = 3, synthetic replicates, N.D.: not detected).

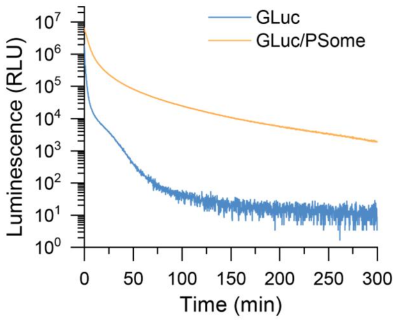

Figure S7. Long-term luminescence kinetics of GLuc (100 ng mL^{-1}) and GLuc/PSomes (adjusted to 100 ng mL^{-1} GLuc) when treated with CTZ.

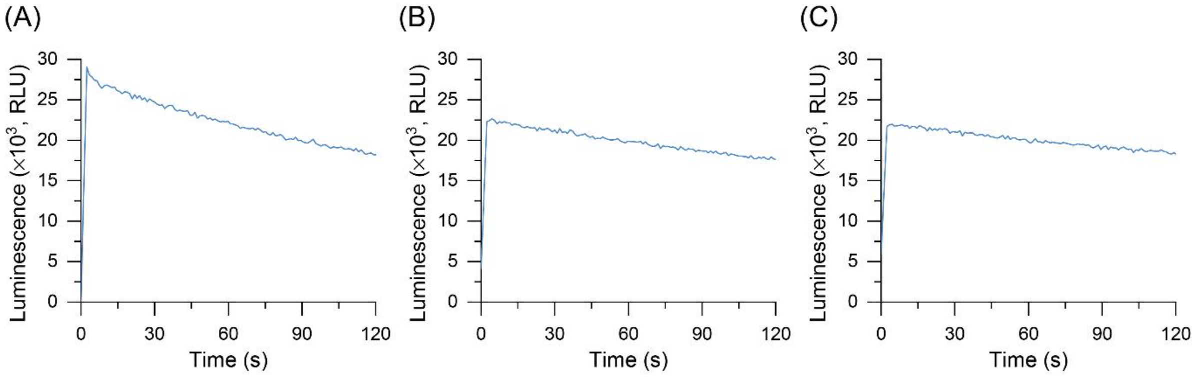

Figure S8. Luminescence kinetics of GLuc/PSomes after **(A)** one, **(B)** two, and **(C)** three repeated treatments with CTZ. This CTZ addition was repeated with 10 min intervals between luminescence measurements.

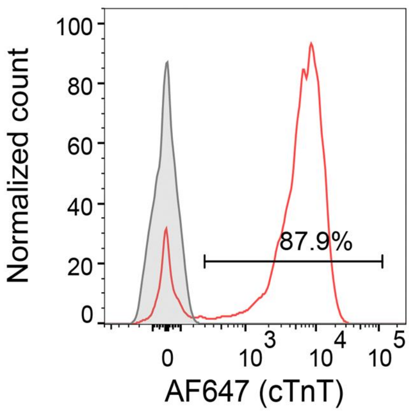

Figure S9. Flow cytometry analysis of Alexa Fluor® 647 (AF647)-conjugated mouse anti-cTnT stained iPSC-CMs after metabolic selection (red). The gray line represents an unstained iPSC-CM control.

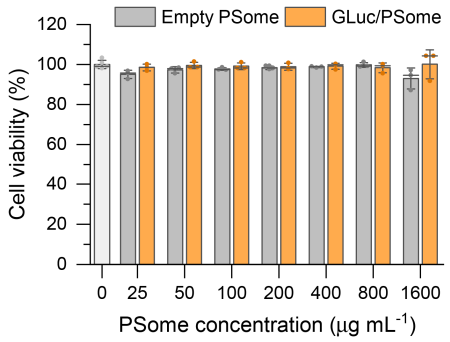

Figure S10. Cell viability of iPSC-CMs after 3 days of incubation with empty PSomes and GLuc/PSomes as determined by PrestoBlue® metabolic assay. The white bar represents the non-treated control (mean ± SD, *n* = 5 for the non-treated control, *n* = 3 for all other PSome-treated cells, technical replicates).

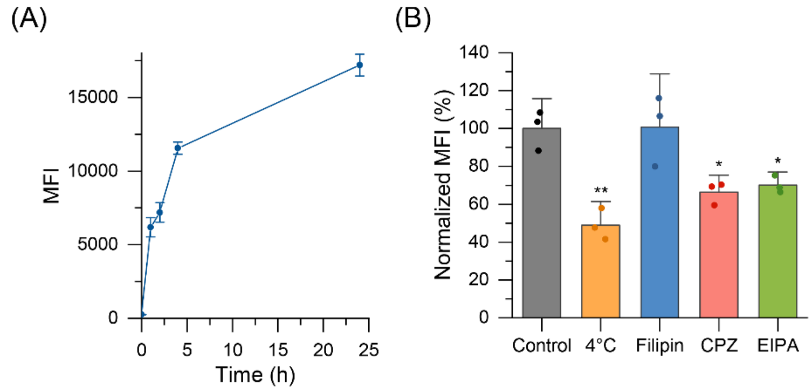

Figure S11. **(A)** Cellular uptake kinetics of GLuc-Cy5/PSomes into iPSC-CMs and **(B)** effects of endocytosis inhibitors on cellular uptake analyzed by quantifying mean fluorescence intensity (MFI) from flow cytometry analysis (mean ± SD, *n* = 3, technical replicates, ***p* < 0.01, **p* < 0.05 vs. control (normal uptake at 37°C) based on one-way ANOVA with Dunnett's multiple comparisons test).

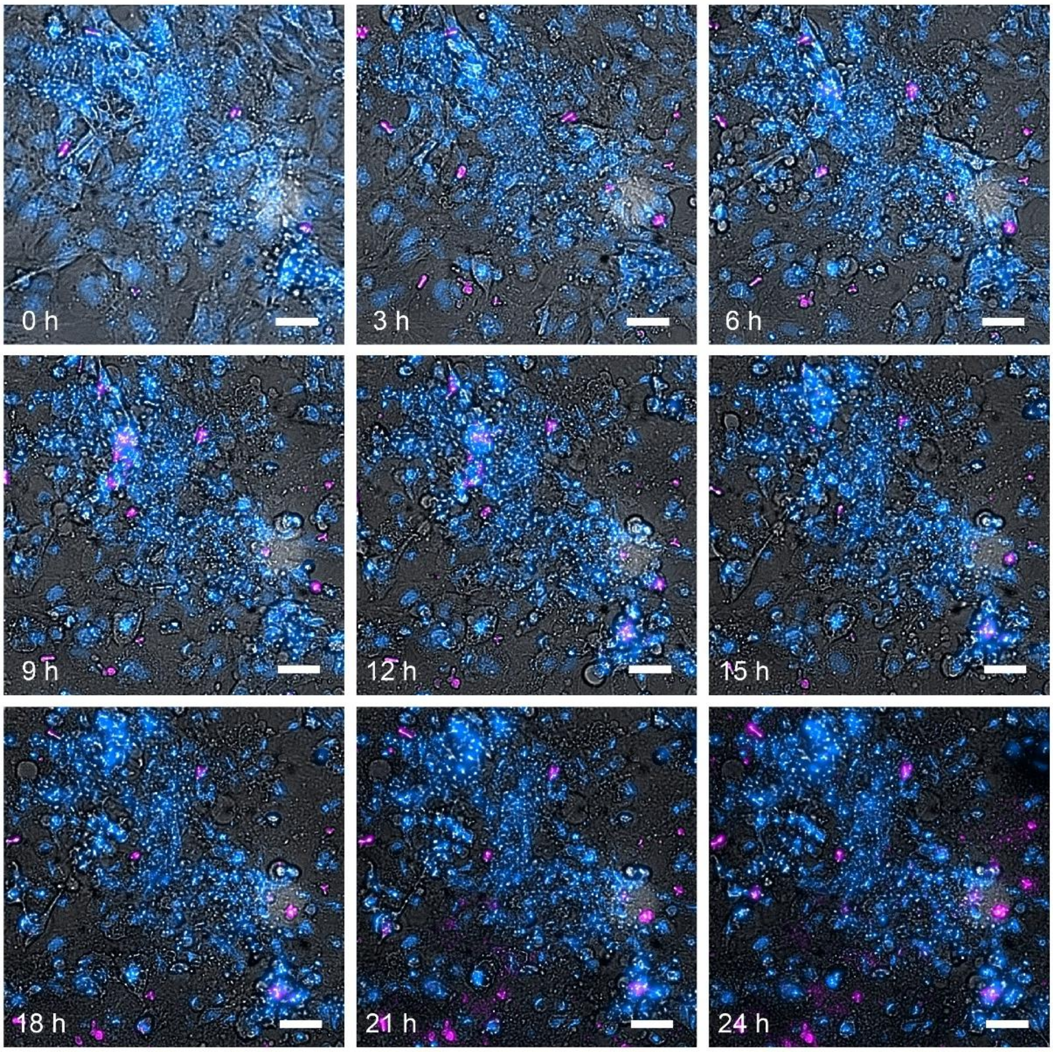

Figure S12. Time-lapse widefield live-cell microscopy images of iPSC-CMs after incubation with GLuc-Cy5/PSomes. The nuclei were stained with Hoechst 33342 (blue) and encapsulated GLuc was labeled with Cy5 (magenta). Scale bar: 50 µm. The images represent the overlay of the two fluorescence channels and the corresponding brightfield image. The full time-lapse containing images acquired every 2 minutes has been compiled in **Supplemental Movie S1**.

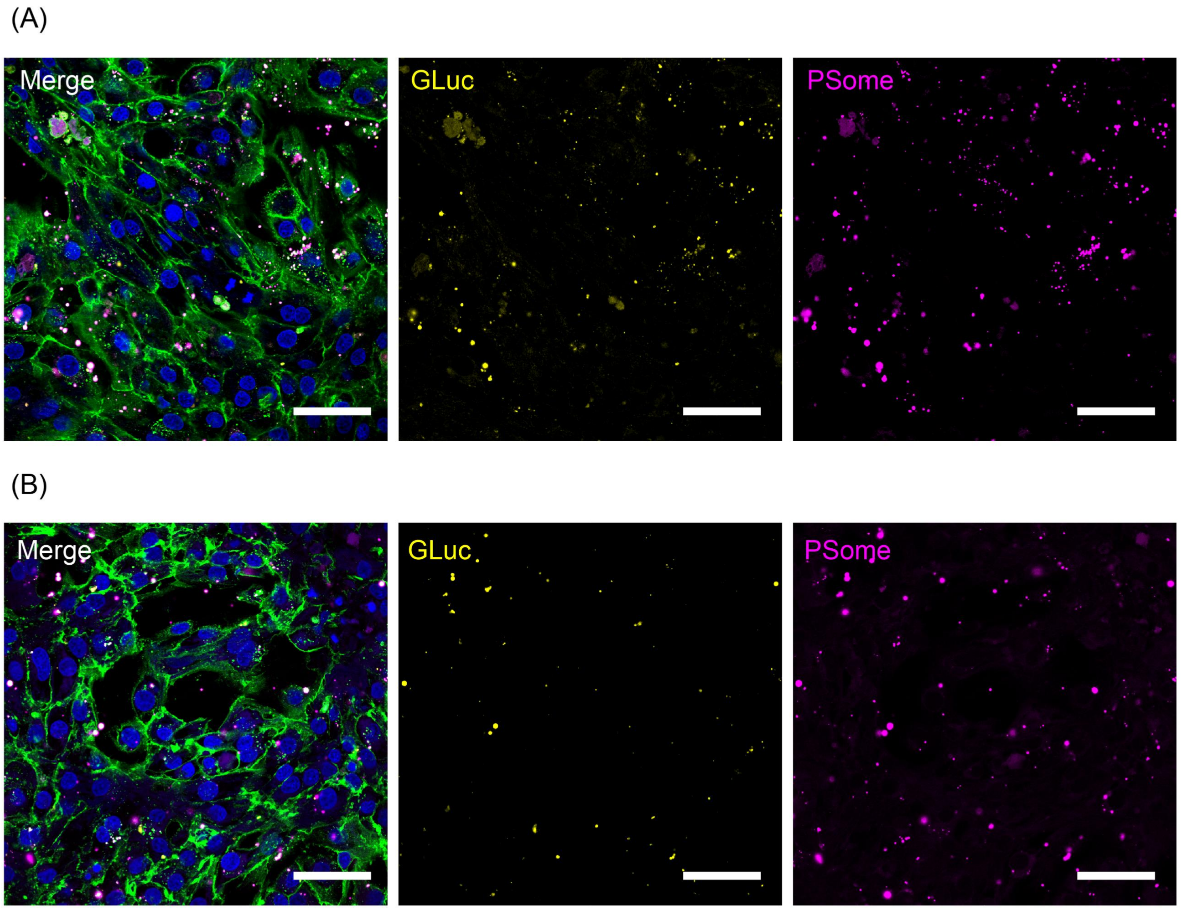

Figure S13. Representative confocal microscopy images of plasma membrane (WGA) stained iPSC-CMs after incubation with **(A)** dual labeled GLuc-Cy5/PSome-RhoB for 24 h or **(B)** dual labeled GLuc-Cy5/PSome-RhoB for 24 h, followed by culturing in maintenance medium for an additional 7 days. The nuclei were stained with DAPI (blue), plasma membrane was stained with AF488-conjugated WGA (green), GLuc with Cy5 (yellow) and the PSome with RhoB (magenta). Scale bar: 50 µm. The merged images represent the overlay of the four separate channels.

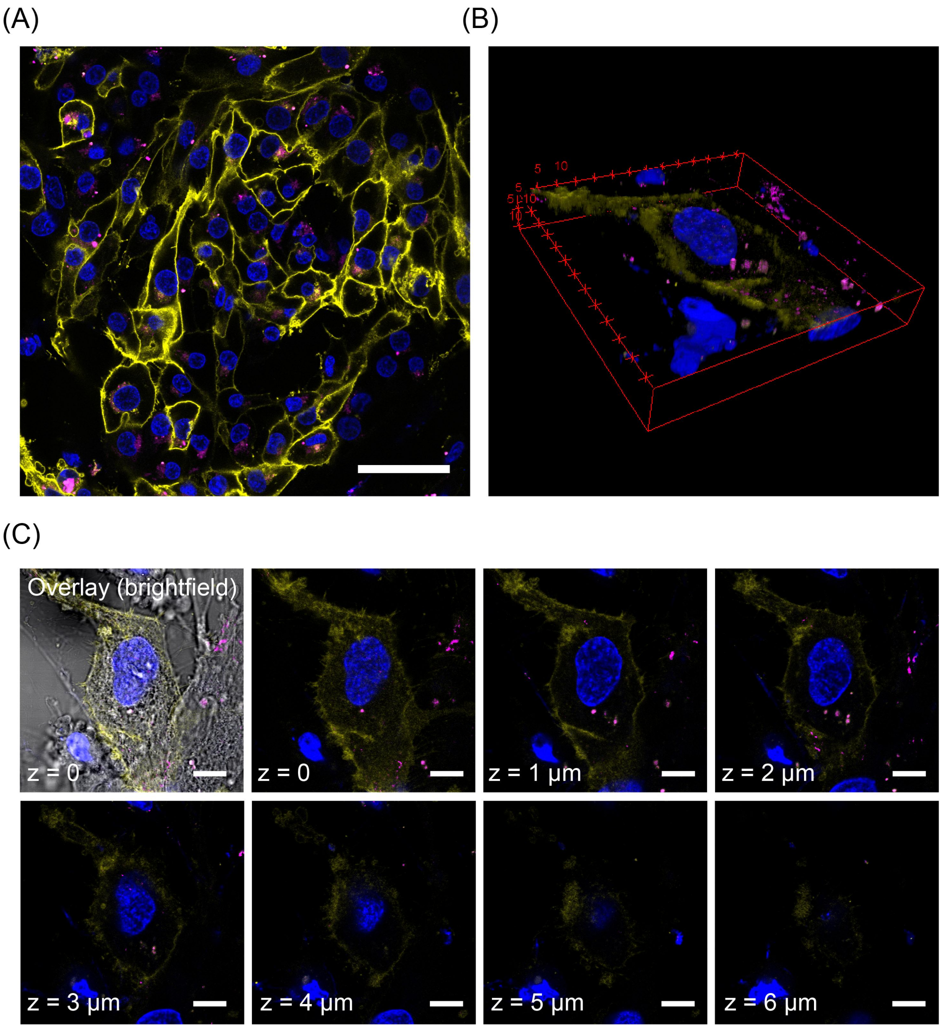

Figure S14. **(A)** Representative confocal microscopy image (scale bar: 50 µm) and **(B)** 3D reconstruction (grid size: 67 × 67 × 11.5 µm) obtained from Z-stack confocal images of iPSC-CMs transduced with ChR2 fused to EYFP as a reporter protein (yellow) and incubated with GLuc-Cy5/PSomes (magenta) for 24 h. The nuclei were stained with Hoechst 33342 (blue). **(C)** Z-stack images (scale bar: 10 µm) were obtained with 0.5 µm steps from the bottom to the top of the cell membrane (every second image is shown). The top left image in **(C)** shows the overlay of the fluorescent channels with a brightfield image to show the morphology and position of cells.

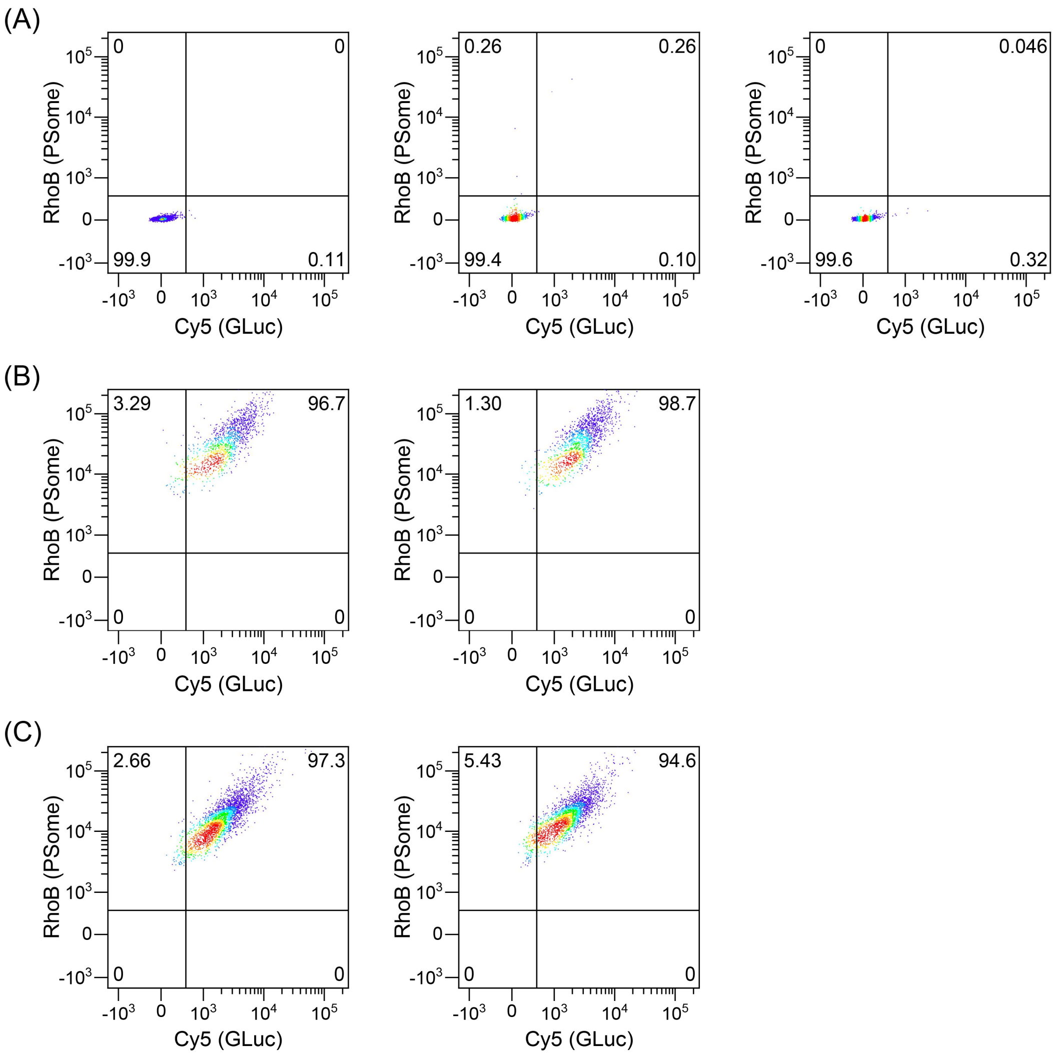

Figure S15. Additional replicates for flow cytometry analysis of iPSC-CMs after incubation with **(A)** blank medium, **(B)** dual labeled GLuc-Cy5/PSome-RhoB for 24 h or **(C)** dual labeled GLuc-Cy5/PSome-RhoB for 24 h, followed by culturing in maintenance medium for an additional 7 days. Representative data are presented in the main text (**Figure 3B and 3E**).

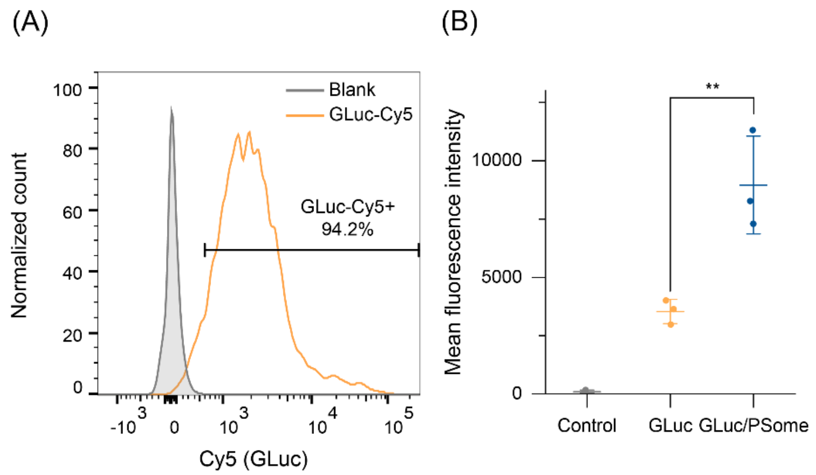

Figure S16. **(A)** Flow cytometry analysis of iPSC-CMs incubated with blank medium or GLuc-Cy5 for 24 h. **(B)** Mean fluorescence intensity obtained from flow cytometry analysis after incubation of iPSC-CMs with blank medium (control), GLuc-Cy5, or GLuc-Cy5/PSome-RhoB for 24 h (mean ± SD, n = 3, technical replicates, **$p < 0.01$ based on one-way ANOVA with Tukey's multiple comparisons test).

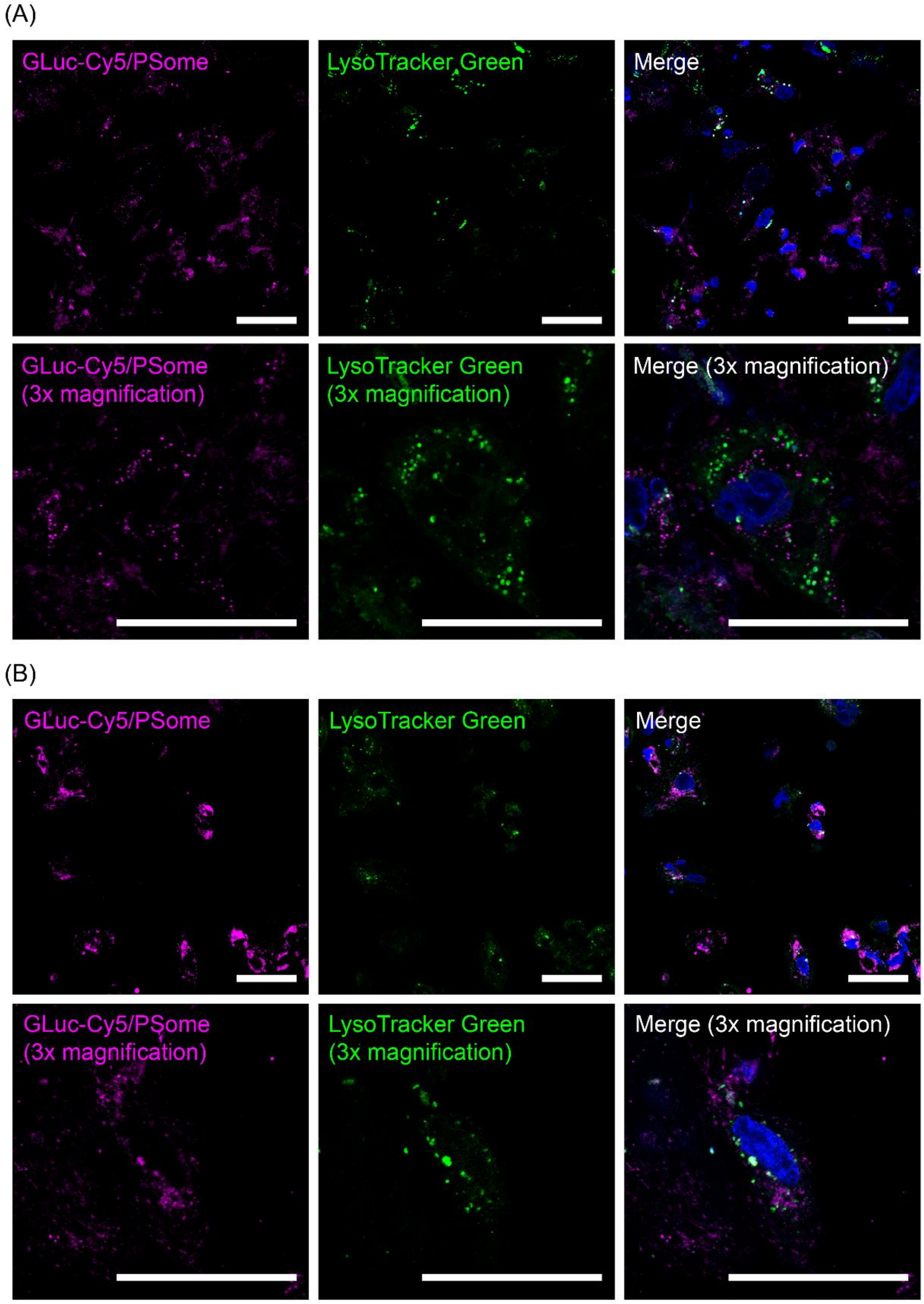

Figure S17. Representative confocal live microscopy images of iPSC-CMs incubated with GLuc-Cy5/PSome for **(A)** 2 h and **(B)** 24 h and stained with LysoTracker™ Green. The nuclei were stained with Hoechst 33342 (blue), lysosomes were stained with LysoTracker™ Green (green), and GLuc was labeled with Cy5 (magenta). Scale bar: 50 µm. The merged images represent the overlay of the three separate channels (the bottom row for each timepoint was acquired with 3× magnification).

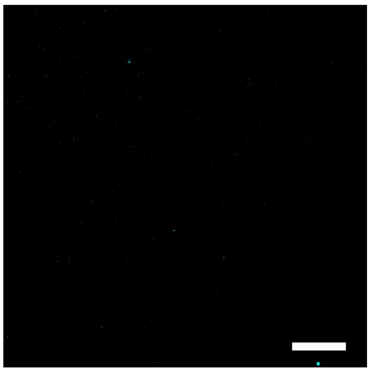

Figure S18. Luminescence microscopy image of control iPSC-CMs (incubated with medium only) after treatment of cells with CTZ. Scale bar: 100 µm.

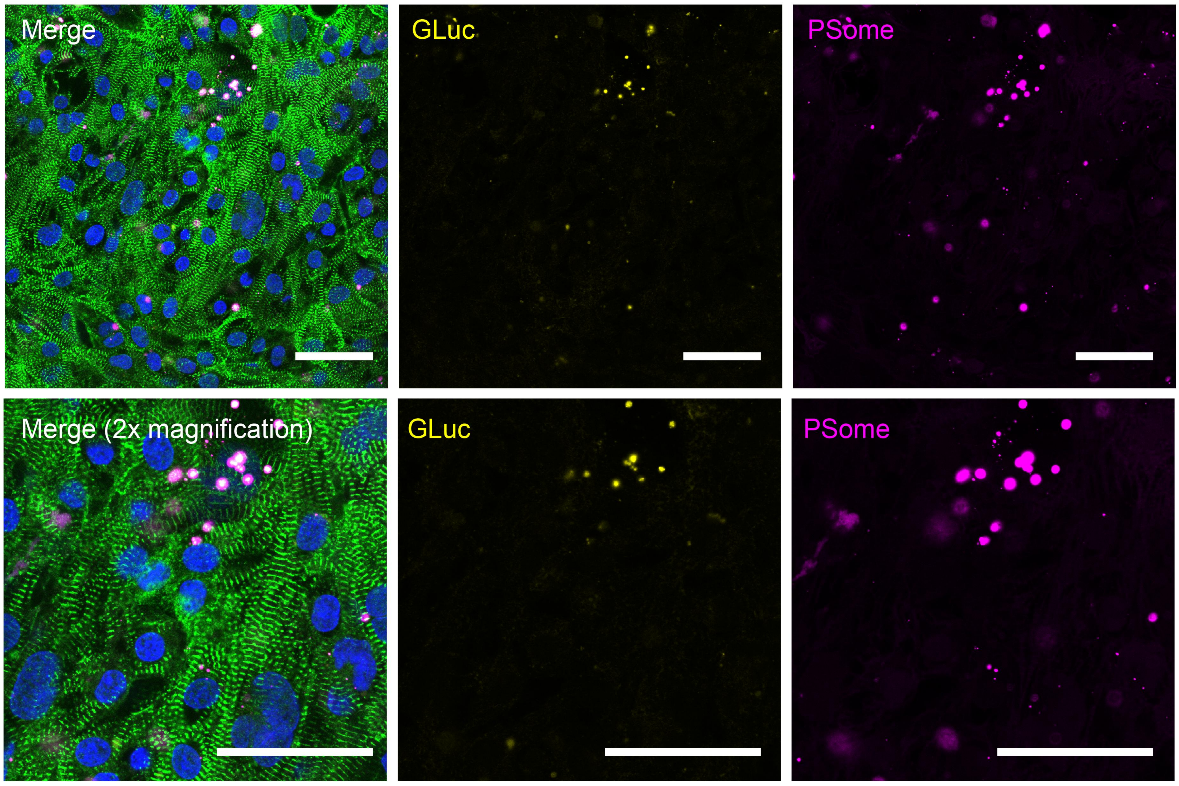

Figure S19. Representative confocal microscopy images of iPSC-CMs cultured for 7 days after incubation with dual labeled GLuc-Cy5/PSome-RhoB for 24 h (the bottom row of images represents a 2× magnification). Nuclei were stained with DAPI (blue), α-actinin was stained with AF488 via a secondary antibody stain (green), GLuc with Cy5 (yellow) and the PSome with RhoB (magenta). Scale bar: 50 µm. The merged images represent the overlay of the four separate channels.

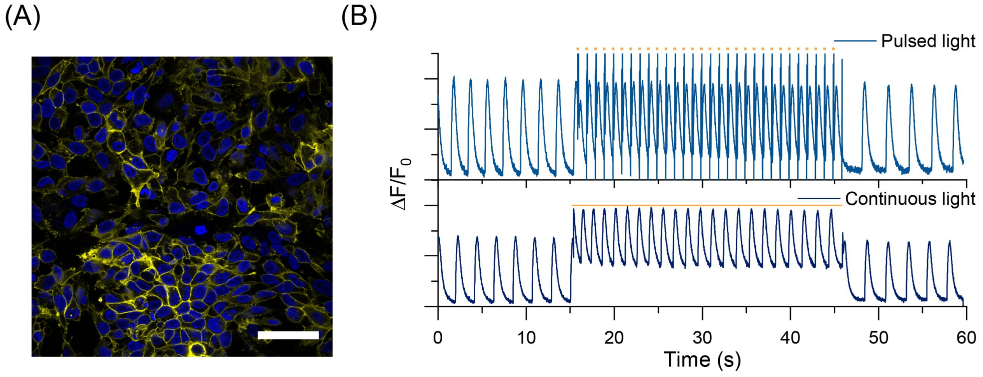

Figure S20. **(A)** Representative confocal microscopy image of iPSC-CMs transduced with ChR2 fused to EYFP as a reporter protein (yellow). Scale bar: 50 µm. The nuclei were stained with DAPI (blue). **(B)** Recordings of calcium transients in ChR2-transduced iPSC-CMs stimulated by a 470 nm LED array with either pulsed light ($I \sim 5.6$ mW cm^{-2}, frequency: 1 Hz, and pulse width: 100 ms) or continuous light ($I \sim 1.6$ mW cm^{-2}). Yellow lines have been added to represent the light stimulation timing. Calcium transients were visualized by staining with Rhod-4 AM prior to imaging.

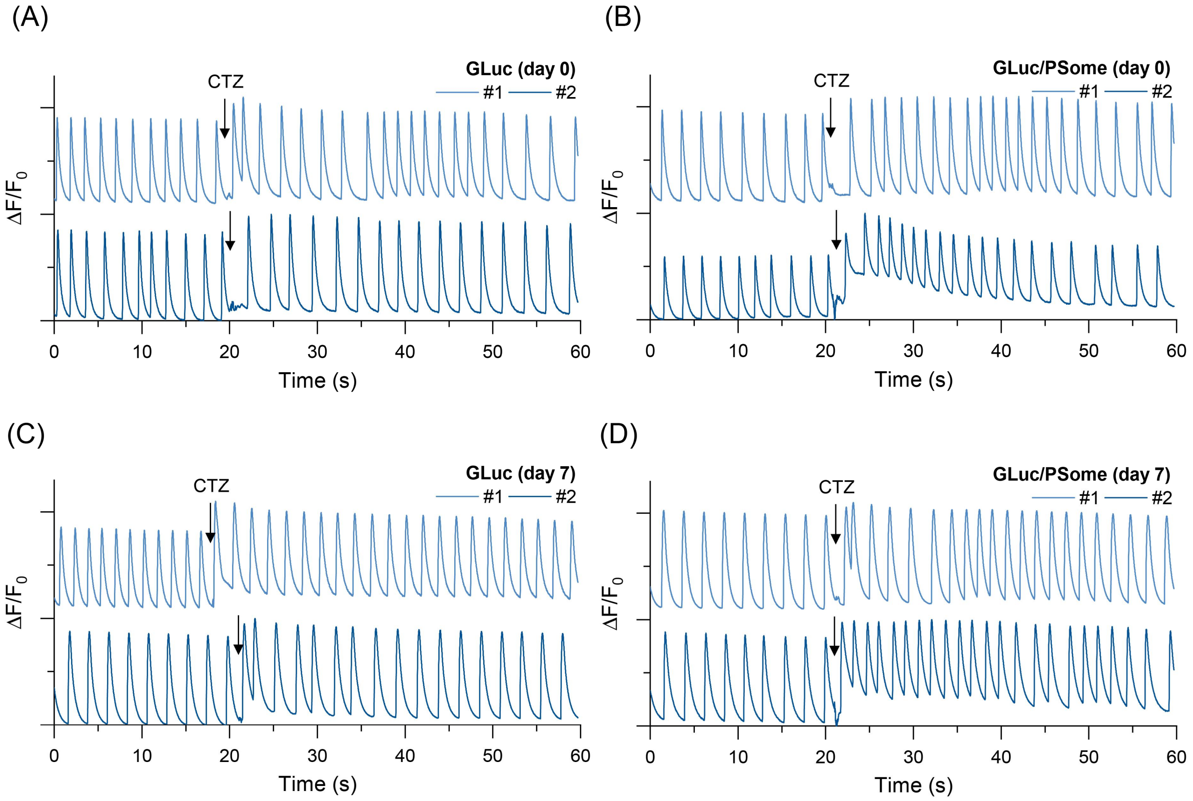

Figure S21. Additional replicates for recordings of calcium transients during CTZ treatment in ChR2-transduced iPSC-CMs after incubation with **(A)** GLuc or **(B)** GLuc/PSome for 24 h, Calcium transients were visualized by staining with Rhod-4 AM prior to imaging. CTZ addition (black arrow) was performed manually after approximately 20 s to record sufficient baseline cardiomyocyte activity. The same imaging experiment was repeated **(C, D)** after culturing the already treated cells in maintenance medium for an additional 7 days. Each experimental condition was repeated in triplicate. Representative data are presented in the main text (**Figure 4B and 4D**).

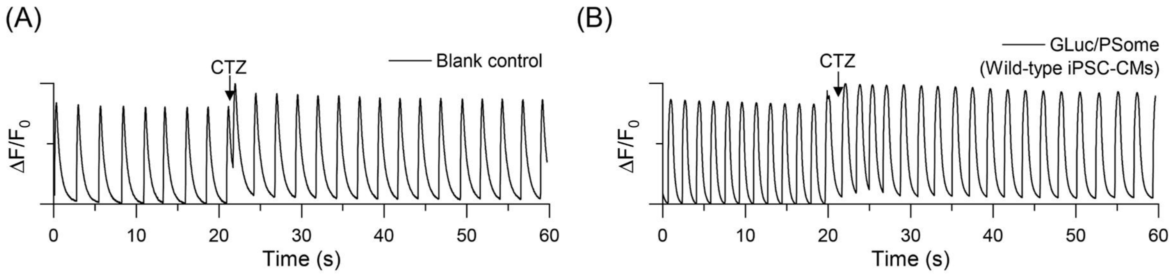

Figure S22. Recordings of calcium transients in response to CTZ stimulation in **(A)** ChR2-transduced iPSC-CMs without any internalized GLuc and **(B)** wild-type iPSC-CMs with internalized GLuc/PSomes. Calcium transients were visualized by staining with Rhod-4 AM prior to imaging. CTZ addition (black arrow) was performed manually after approximately 20 s to record sufficient baseline cardiomyocyte activity.

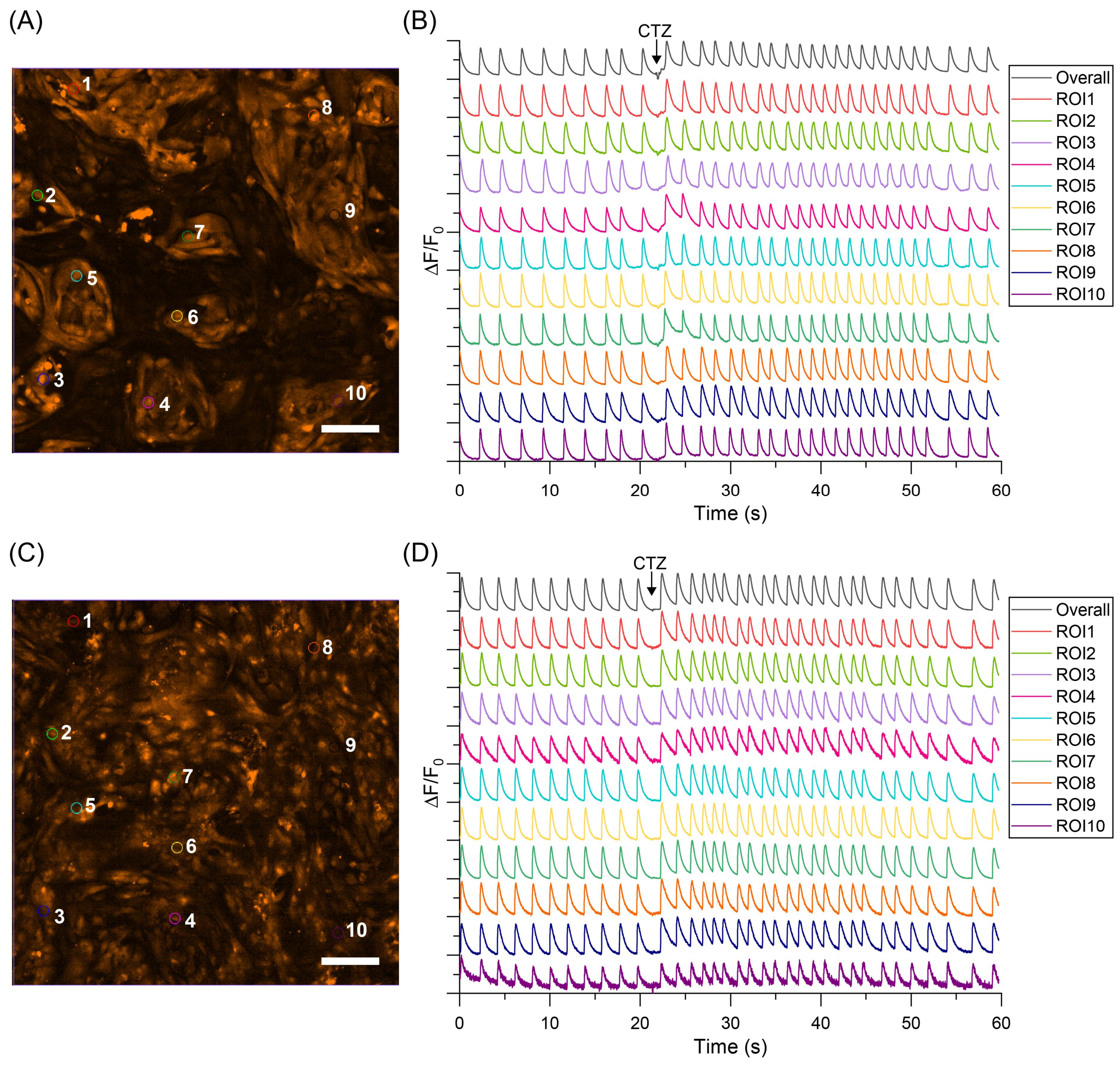

Figure S23. **(A,C)** Fluorescence microscopy images of ChR2-transduced iPSC-CMs with internalized GLuc/PSomes. ROIs used for the image analysis are marked with circles. **(B,D)** Recordings of calcium transients over time during CTZ treatment are separately plotted for each ROI as well as the overall image intensity of each frame. Data contained in **(A,B)** were obtained during the CTZ treatment of ChR2-transduced iPSC-CMs with uptaken GLuc/PSomes on day 0 after uptake (**Figure 4B**) and **(C,D)** were obtained during a second CTZ treatment after an additional 7 days in culture (**Figure 4D**). Scale bar: 200 µm.

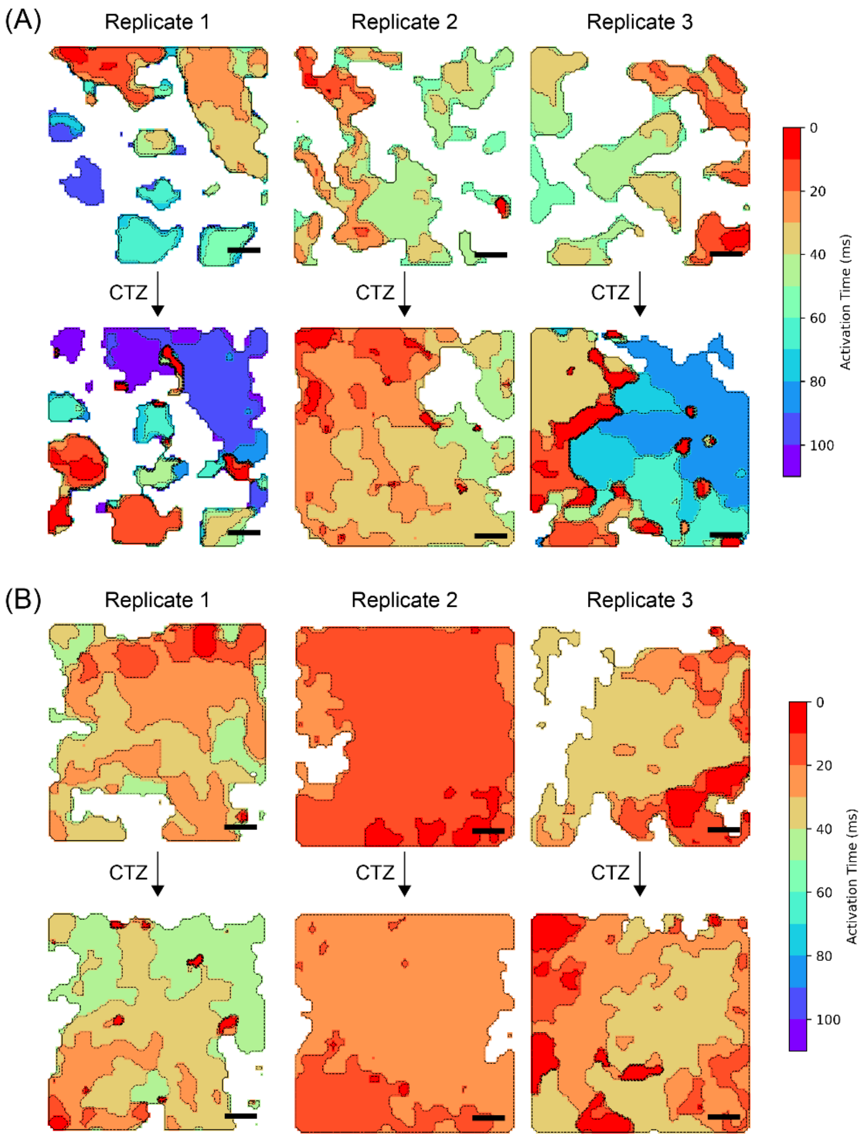

Figure S24. Activation maps before and after treatment with CTZ in ChR2-transduced iPSC-CMs with internalized GLuc/PSomes on **(A)** day 0 and **(B)** day 7 (three replicates per group). Scale bar: 200 µm.

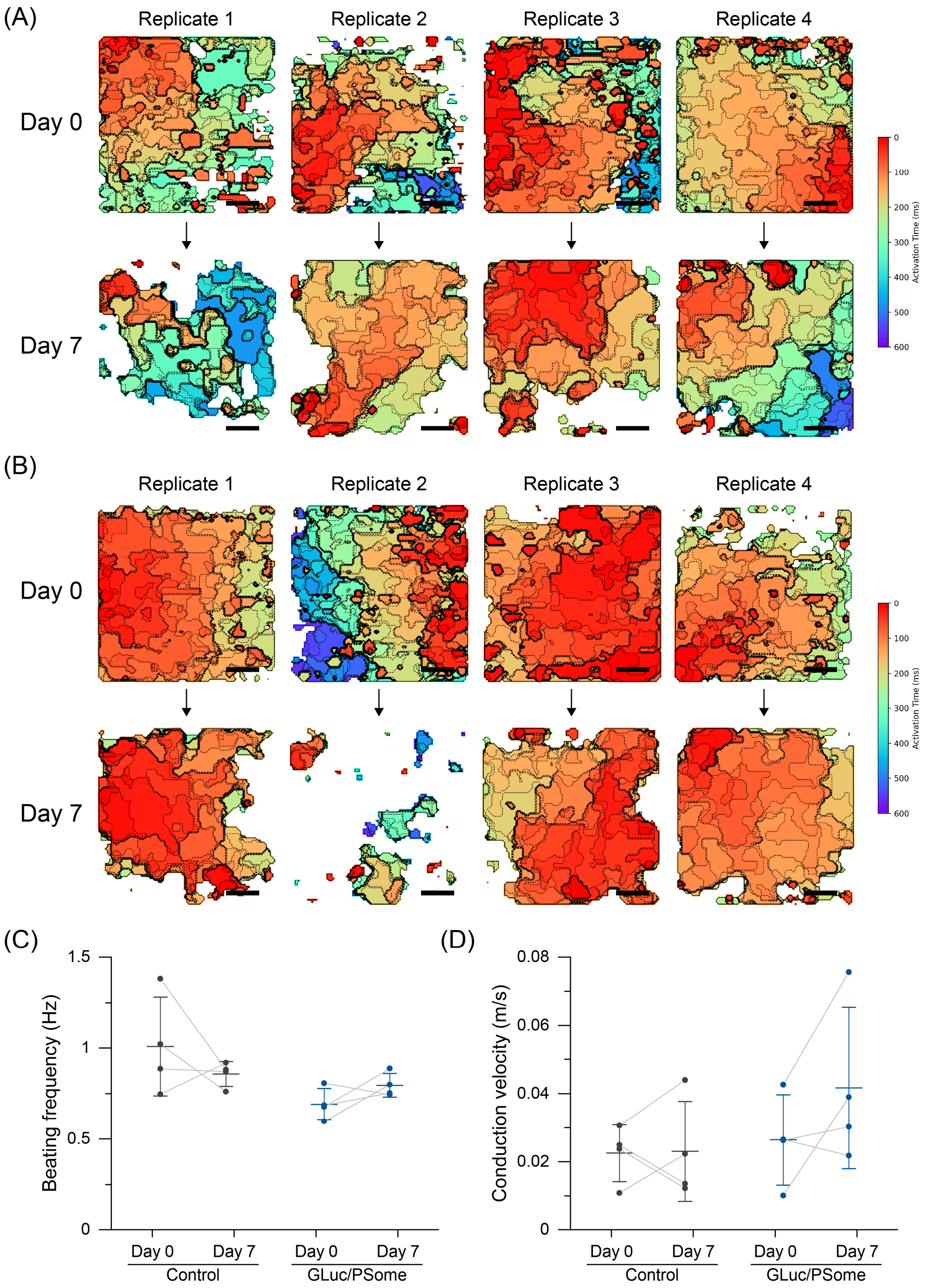

Figure S25. **(A,B)** Activation maps of iPSC-CMs on day 0 and day 7 after incubation with **(A)** blank medium or **(B)** GLuc/PSomes for 24 h (four replicates each group). Scale bar: 1 mm. **(C)** Beating frequency and **(D)** conduction velocity changes during the culture of iPSC-CMs for 7 days after incubation with blank medium (control) or GLuc/PSomes for 24 h (mean ± SD, n = 4). The conduction velocities in **(D)** were calculated based on **(A,B)**.

Supplemental Movie 1. Time-lapse widefield live-cell microscopy video of iPSC-CMs after incubation with GLuc-Cy5/PSomes. The nuclei were stained with Hoechst 33342 (blue) and encapsulated GLuc was labeled with Cy5 (magenta). The images represent the overlay of the two fluorescence channels and the corresponding brightfield image. Elapsed time is shown in the bottom left corner (hh:mm).

Supplemental Movie 2. Luminescence microscopy video of **(A)** iPSC-CMs and **(B)** iPSC-CMs with internalized GLuc/PSomes. CTZ was added 2 s after the start of the recording.

Supplemental Movie 3. Brightfield microscopy video of ChR2-transduced iPSC-CMs with internalized GLuc/PSomes in response to the addition of CTZ. CTZ addition was performed manually after approximately 15 s to record sufficient baseline cardiomyocyte activity.

Supplementary References

[1] P. Müller, P. Schwille, T. Weidemann, *Bioinformatics* **2014**, *30*, 2532.
[2] P. Kapusta, *Absolute Diffusion Coefficients: Compilation of Reference Data for FCS Calibration*, PicoQuant GmbH, 2010.
[3] K. Bacia, S. A. Kim, P. Schwille, *Nat. Methods* **2006**, *3*, 83.
[4] S. Xu, J. Yeow, C. Boyer, *ACS Macro Lett.* **2018**, *7*, 1376.
[5] C. Gao, X. Zheng, *Soft Matter* **2009**, *5*, 4788.
[6] F. Zhang, V. Gradinaru, A. R. Adamantidis, R. Durand, R. D. Airan, L. de Lecea, K. Deisseroth, *Nat. Protoc.* **2010**, *5*, 439.
[7] P. W. Burridge, E. Matsa, P. Shukla, Z. C. Lin, J. M. Churko, A. D. Ebert, F. Lan, S. Diecke, B. Huber, N. M. Mordwinkin, J. R. Plews, O. J. Abilez, B. Cui, J. D. Gold, J. C. Wu, *Nat. Methods* **2014**, *11*, 855.
[8] T. Grune, C. Ott, S. Häseli, A. Höhn, T. Jung, *Sci. Rep.* **2019**, *9*, 15112.
